# A Brain Penetrant Mutant IDH1 Inhibitor Provides *In Vivo* Survival Benefit

**DOI:** 10.1038/s41598-017-14065-w

**Published:** 2017-10-23

**Authors:** Johnny Kopinja, Raquel S. Sevilla, Diane Levitan, David Dai, Amy Vanko, Edward Spooner, Chris Ware, Robert Forget, Kun Hu, Astrid Kral, Peter Spacciapoli, Richard Kennan, Lata Jayaraman, Vincenzo Pucci, Samanthi Perera, Weisheng Zhang, Christian Fischer, Michael H. Lam

**Affiliations:** Merck & Co., Inc., Merck Research Laboratories, Kenilworth, NJ 07033 USA

## Abstract

Mutations in IDH1 are highly prevalent in human glioma. First line treatment is radiotherapy, which many patients often forego to avoid treatment-associated morbidities. The high prevalence of IDH1 mutations in glioma highlights the need for brain-penetrant IDH1 mutant-selective inhibitors as an alternative therapeutic option. Here, we have explored the utility of such an inhibitor in IDH1 mutant patient-derived models to assess the potential therapeutic benefits associated with intracranial 2-HG inhibition. Treatment of mutant IDH1 cell line models led to a decrease in intracellular 2-HG levels both *in vitro* and *in vivo*. Interestingly, inhibition of 2-HG production had no effect on *in vitro* IDH1 mutant glioma cell proliferation. In contrast, IDH1 mutant-selective inhibitors provided considerable survival benefit *in vivo*. However, even with near complete inhibition of intratumoral 2-HG production, not all mutant glioma models responded to treatment. The results suggest that disruption of 2-HG production with brain-penetrant inhibitors in IDH1 mutant gliomas may have substantial patient benefit.

## Introduction

Mutations in the *isocitrate dehydrogenase 1* (*IDH1*) gene have been observed in multiple human tumor types, with the highest prevalence seen in low grade glioma (LGG) and secondary glioblastoma (GBM)^1–4^. LGG primarily affects younger patients, with a median age of approximately 35 years^4^. LGG patients do not appear to respond well to standard aggressive therapy^5^. A common subtype of LGG, astrocytoma, have been shown to be 70% IDH1 mutant^4^. Patients with glioma have a decreased quality of life and lifespan, many eventually succumbing to disease. These findings demonstrate there is a strong unmet medical need for targeted mutant-selective therapies for the treatment of patients with IDH1 mutant gliomas.

In glioma patients, mutations in IDH1 typically occur at the arginine residue located at amino acid position 132 resulting in several protein variants. A histidine (R132H) alteration is the most frequently observed amino acid alteration in these patients^4^. These mutations lead to a neomorphic gain of function, and enzymatic overproduction, resulting in high intratumoral levels of 2-hydroxyglutarate (2-HG)^6^. High 2-HG levels have been shown to inhibit dioxygenase enzymes involved in regulating epigenetic mechanisms associated with histone and DNA methylation thereby promoting tumorigenesis^7^. The epigenetic dysregulation imparted by such IDH1 mutations can be clearly observed by the global hypermethylation phenotypes observed in tumor types bearing these alterations^8,9^. Specifically, in glioma patients, IDH1 mutant tumors stratify into a distinct subgroup of gliomas displaying a pronounced CpG island methylator phenotype (G-CIMP)^9^. Xu *et al*. have shown that elevated levels of 5-methylcytosine found in IDH1 mutant cells can be reversed with the removal of 2-HG^7^. This finding suggests that pharmacologic inhibitors of 2-HG production may alter the epigenetic and, ultimately, the gene expression profile of these mutant tumors.

As per The Cancer Genome Atlas (TCGA) Research Network, the proneural molecular subgroup of glioblastoma encompasses tumors which harbor IDH1 mutations and alterations in *PDGFRA* ^5^. This signature is comprised of proneural development genes, such as *DLL3* and *SOX2*, in addition to the oligodendrocytic development genes *PDGFRA*, *NKX2.2* and *OLIG2* ^5,10^. Given the minimal number of IDH1 mutant tumor samples in the TCGA dataset, Cooper *et al*. examined and confirmed the expression of this proneural signature in lower grade gliomas, which are known to be highly enriched for IDH1 mutations^5,10^. These findings suggest that IDH1 mutant tumors are indeed strongly associated with the proneural gene expression subtype observed in patients with gliomas.

Patient-derived *in vivo* models for the preclinical study of IDH1 mutant glioma are scarce. New IDH1 mutant glioma models are needed to examine the spectrum of responses to treatment that may be observed clinically following the administration of IDH1 mutant inhibitors. Luchman *et al*. characterized the BT142 IDH1 R132H mutant model as having multi-lineage potential for its ability to express either astrocytic or oligodendrocytic markers depending on the *in vitro* stimulus^11^. This cell line produces high levels of 2-HG and is capable of growing intracranially in immuno-compromised mice. BT142 cells have been shown to divide very slowly *in vitro* when maintained as a neurosphere culture. However, upon intracranial implantation in the striatum of mice, BT142 aggressively proliferates resulting in animal mortality after approximately 12 weeks. Importantly, BT142 maintains mutant IDH1 and wildtype heterozygosity when serially propagated *in vivo*. This finding suggests the *in vivo* proliferative capacity of this model is dependent on a yet-to-be defined tissue microenvironment. Further analysis revealed that BT142 displays an undifferentiated glial cell state, defined as lacking the expression of several glial-associated cell markers. This finding suggests the BT142 cell line displays properties of brain tumor stem cells and, as such, is consistent with the theory that high levels of 2-HG block cellular differentiation^12–15^. These findings suggest that treatment with a mutant IDH1 inhibitor may confer a meaningful survival benefit in BT142 inoculated mice.

In this report, we describe the effects of a brain-penetrant small molecule inhibitor of mutant IDH1 on *in vitro* and *in vivo* 2-HG production in mutant cell lines and patient-derived orthotopic glioma xenograft mouse models.

## Results

### Discovery of mutant-selective IDH1 inhibitors and *in vitro* response in IDH1 mutant cell lines

**Figure 1:**
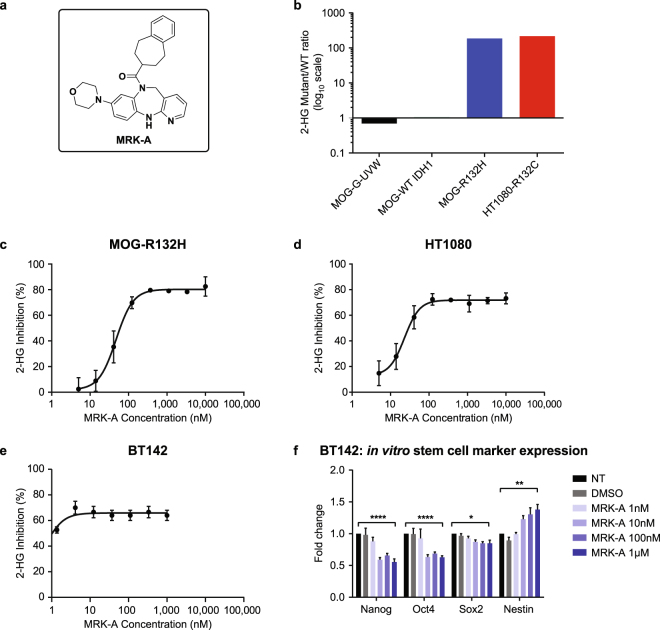
(**a**) Chemical structure of MRK-A. (**b**) MOG-G-UVW, MOG-R132H and HT1080 2-HG levels at baseline. MOG-G-UVW was engineered to express either wildtype (MOG-WT IDH1, black) or mutant IDH1 R132H (MOG-R132H, blue). HT1080 IDH1 R132C fibrosarcoma cells (red) is shown as a reference for 2-HG production. (**c–e**) *In vitro* curves for 2-HG inhibition by MRK-A in MOG-R132H, HT1080, and BT142 cell lines. Inhibition curves for (**c**) MOG-R132H, (**d**) HT1080, and (**e**) BT142 displayed in nanomolar (nM). (**f**) MRK-A *in vitro* dose response with BT142 IDH1 R132H glioma cells over 1 week. MRK-A treatment leads to stem cell marker changes in BT142. Stem cell marker expression was examined by qPCR assay. *, **, and **** indicate statistical significance at *P* < 0.05, 0.01, and 0.0001 respectively, compared to vehicle control.

Employing an extensive compound library, we screened for and discovered several IDH1 mutant-selective compounds. We optimized several structurally distinct chemotypes which selectively inhibit the mutant IDH1 enzyme. The compound MRK-A was derived from one of these tractable chemotypes (Fig. 1a). Our screening approach, assay details, and selection criteria are discussed in the methods section; additional details and a full account of the medicinal chemistry strategy will be published at a later time. With a biochemical IC_50_ value of 5 nM, MRK-A potently inhibits mutant IDH1 from generating 2-HG. This compound is highly selective for mutant IDH1, as MRK-A does not inhibit wildtype IDH1 at concentrations below 50,000 nM. This represents a 10,000-fold mutant to wildtype selectivity ratio for MRK-A. Additionally, we investigated whether MRK-A is brain penetrant in naïve animals and the unbound brain to blood ratio was determined to be >1 which is indicative of passive brain penetration. Additional details are provided in the methods section.

To better understand the relationship between 2-HG production and MRK-A inhibition, the compound was tested in both engineered and naturally occurring IDH1 mutant cell lines. Using lentiviral transduction, we induced exogenous mutant IDH1 R132H protein expression in the wildtype IDH1 glioma cell line, MOG-G-UVW (MOG-R132H). We confirmed expression of the mutant protein in this cell line using an IDH1 R132H-specific antibody^16^. As expected, IDH1 R132H expression led to high millimolar levels of intracellular 2-HG production resulting in a greater than 180-fold increase in 2-HG as compared with the parental MOG-G-UVW or the MOG-G-UVW IDH1 wildtype transfected (MOG-WT) lines (Fig. 1b). For comparison, the HT1080 cell line, which harbors an endogenous IDH1 R132C mutation, produces >200-fold more 2-HG than the wildtype cell lines mentioned above (Fig. 1b). Employing MRK-A, we generated cellular 2-HG IC_50_ values for both glioma MOG-R132H and BT142 lines as well as the HT1080 R132C human fibrosarcoma line. As shown in Fig. 1c–e, *in vitro* treatment with MRK-A induced potent inhibition of 2-HG production irrespective of the IDH1 mutant line tested. The much lower IC_50_ value for BT142 is likely correlated with protein binding of MRK-A, as BT-142 is maintained in serum free media, in contrast to 10% FBS with MOG-R132H and HT1080. Thus, MRK-A was determined to be a potent and selective IDH1 mutant inhibitor capable of suppressing 2-HG production in both naturally occurring and mutant overexpressing cell lines.

Next, we used MRK-A to examine the anti-tumor properties associated with *in vitro* 2-HG inhibition in patient-derived mutant IDH1 cell lines. Using MRK-A at concentrations that should completely inhibit 2-HG production, no changes in cell viability were observed in BT142, HT1080 or GB10 (Supplementary Fig. S1a–c) cell lines following treatment with concentrations up to 1 μM. It is important to note that the BT142 cell line does not proliferate well in culture^11^, which limited our ability to assess growth inhibition following MRK-A treatment. GB10 is a novel and proprietary patient-derived IDH1 R132H mutant glioma model developed and characterized below.

Given recent findings that high tumor 2-HG levels have been shown to block cellular differentiation in a variety of IDH1 mutant tumor types, we next examined the ability for MRK-A to alter glioma stem cell marker expression in BT142^12–15,17^. One such marker is the intermediate filament protein, nestin, which has been extensively studied in human glioma and neural stem cells^18–20^. After 1 week of MRK-A treatment *in vitro*, BT142 cells displayed a modest, but statistical increase in nestin expression (Fig. 1f). This finding contrasts with previous findings where nestin downregulation was observed in studies which employed both a different mutant IDH1 inhibitor in an oligodendroglioma model^17^. We eventually examined nestin levels *in vivo* post-MRK-A treatment and found no statistical difference between vehicle and drug treated orthotopic tumors (Supplementary Fig. S3e,f). To explore this *in vitro* phenotype further, we also examined a number of other glial lineage markers including GFAP, GALC and CNP, but found no change in the expression of these genes with MRK-A treatment (Supplementary Fig. S1d–f). Given the previous characterization of BT142 cells as an undifferentiated glial tumor initiating line^11^, we extended the analysis to include the master pluripotency regulators, Nanog, Sox2 and Oct4. Over the course of one week of *in vitro* MRK-A treatment, we observed substantial downregulation of Nanog and Oct4 in BT142 cells at concentrations as low as 10 nanomolar (Fig. 1f). Additionally, we also detected a decrease in Sox2, however to a lesser extent than that found for Nanog and Oct4 (Fig. 1f). With an IC_50_ of ~5 nM for MRK-A in BT142, the concentrations used here are predicted to induce substantial and prolonged *in vitro* 2-HG inhibition which led to marked changes in key stem cell markers. In the BT142 IDH1 mutant glioma line, these data suggest that 2-HG inhibition may lead to changes in cell differentiation.

### IDH1 mutant glioma model development and *in vivo* analysis of 2-HG inhibition

#### Imaging of GB10 and BT142 and correlation of luciferase signal to MRI

**Figure 2:**
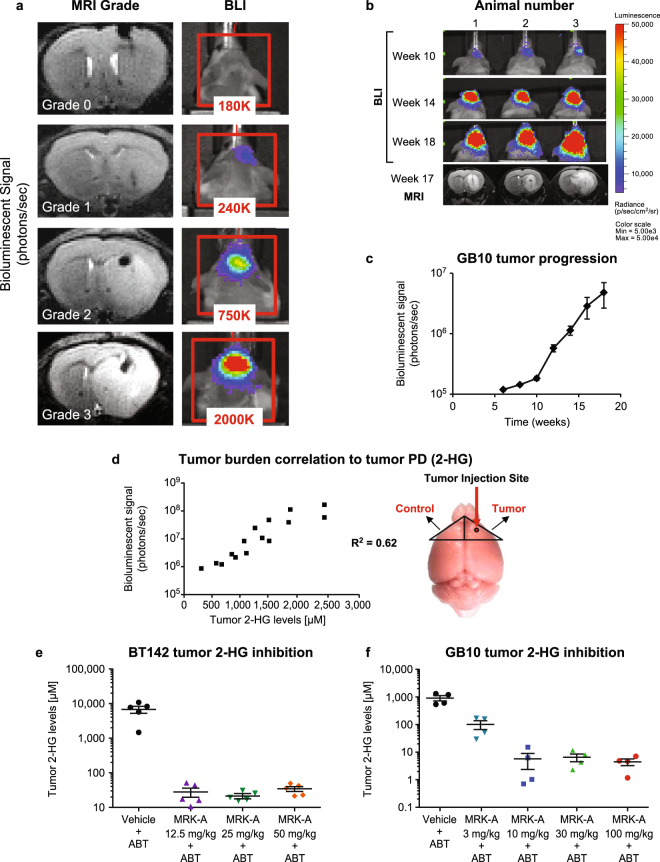
(**a**) BT142-luciferase characterization and tumor burden assessment using Bioluminescence and MRI. Mice were injected intracranially with a patient-derived glioma cell line engineered to express luciferase protein. Representative anatomical MR images and corresponding bioluminescent heat maps are shown. On the left, the MR images were graded based on tumor size and intensity using a scoring system ranging from 0 = no tumor to 5 = very large, hyperintense tumor invading the brain hemisphere contralateral to the injection site. On the right, the heat map represents emitted photons per second. (**b**) GB10-luciferase characterization and tumor burden assessment versus MRI. GB10, a novel, patient-derived tumor model was engineered to express luciferase protein. Representative images show increasing bioluminescent signal over time. MR images at week 17 are displayed for comparative purposes and demonstrate that GB10 presents as a discrete mass instead of a hyperintense and diffuse tumor as in BT142. (**c**) GB10 tumor progression was measured as increasing intensity in bioluminescent signal over time. (**d**) Bioluminescent signal correlates to 2-HG levels in BT142 tumor model. *In vivo* LC-MS-based detection of 2-HG in BT142-injected animals with increasing tumor burden as indicated by bioluminescent imaging. The mouse brain on the right shows the approximate location of tumor cell injection (red arrow pointing to circle). (**e,f**) MRK-A dose response in patient-derived IDH1 mutant BT142 and GB10 models. For both studies, BID dosing occurred over three days, for a total of five doses. (**e**) Tumor 2-HG inhibition following three BID doses of 12.5, 25 and 50 mg/kg of MRK-A, administered orally in the BT142 tumor model. (**f**) Inhibition of 2-HG production in tumors following three oral doses of MRK-A, at 3, 10, 30 or 100 mg/kg, in the GB10 orthotopic glioma model.

Given the contextual microenvironment in which tumor stem cells may proliferate, we sought to understand the potential therapeutic benefit of treating IDH1 mutant glioma cell lines *in vivo*. To assess glioma growth intracranially and relate tumor burden to 2-HG levels, we had to devise a method to effectively monitor disease progression with 2-HG production *in vivo*. Therefore, we engineered the expression of luciferase in BT142 (BT142-luc) and GB10 (GB10-luc). To characterize these *in vivo* imaging models, we tracked tumor progression by magnetic resonance imaging (MRI) to ensure reliable correlation of luciferase signal to physical tumor progression for BT142 (Fig. 2a); we also examined tumor growth by BLI for GB10 correlating with MRI scoring at week 17/18 (Fig. 2b) and characterized the full growth curve by BLI to understand tumor growth kinetics (Fig. 2c). We used one of two methods to quantify tumor burden from the MR images. BT142 tumors show a diffuse growth pattern so tumors are graded based on severity. Unlike the BT142 model, GB10 tumors present as a discrete mass, therefore, tumor volumes were segmented instead of graded. MR imaging allowed the tracking of tumor volumes and provided a visual reference to monitor tumor progression which we describe further below. Bioluminescence tracked well with anatomical MR imaging in these two intracranial glioma models.

#### Tumor burden correlation to 2-HG production

These imaging approaches also enabled our understanding of the relationship between tumor burden and 2-HG production *in vivo*. By incorporating these imaging techniques and careful resectioning of glioma tissue from affected brain regions, we were able to robustly monitor tumor 2-HG production by LC-MS. Tumors were monitored until they reached a suitable size before harvest, such that we could more accurately measure 2-HG *ex vivo*. As shown, for BT142 we were able to correlate increasing luciferase signal to increasing intracranial glioma 2-HG levels (Fig. 2d). Bioluminescent imaging of tumor burden correlated well to 2-HG production with an R-squared value of 0.62. Our efforts to surgically implant and carefully develop techniques to monitor tumor progression and *in vivo* 2-HG production allowed us to examine pharmacological inhibition of 2-HG production in these models.

#### MRK-A inhibits 2-HG production in both BT142 and GB10 intracranial xenografts

After characterization of these models, we conducted studies to understand intracranial small molecule-based 2-HG inhibition with MRK-A. Using the imaging techniques described above, we assembled either BT142- or GB10-inoculated animals into cohorts of similar tumor burden. In addition to administering a range of MRK-A doses, we increased compound exposure by co-dosing MRK-A with the CYP450 inhibitor aminobenzotriazole (ABT) (Fig. 2e,f). In order to assess steady-state pharmacokinetics and 2-HG inhibition we conducted our PKPD experiments by administering 5 bis in die (BID) doses of MRK-A with ABT and sacrificed the animals on day 3.The treatment of BT142 animals with MRK-A resulted in near complete inhibition (>99%) of intracranial tumor 2-HG production across the 12.5, 25, and 50 mg/kg + ABT 50 mg/kg BID dose groups, respectively. In GB10 animals, using the same BID dosing regimen + ABT 50 mg/kg BID, robust inhibition of 2-HG production was observed across all doses tested: 3, 10, 30, and 100 mg/kg. All doses tested achieved >99% inhibition of intratumoral 2-HG production *in vivo*, except the 3 mg/kg group, which inhibited 2-HG up to 88% (Fig. 2e,f). Given the robust inhibition of 2-HG production observed across these MRK-A doses, the MRK-A 30 mg/kg dose was selected for all future studies described herein. These data show that MRK-A achieves prolonged intracranial 2-HG inhibition in patient-derived glioma models.

### Efficacy studies in BT142, U87MG and GB10 glioma models

#### MRK-A induces tumor growth inhibition in BT142

**Figure 3:**
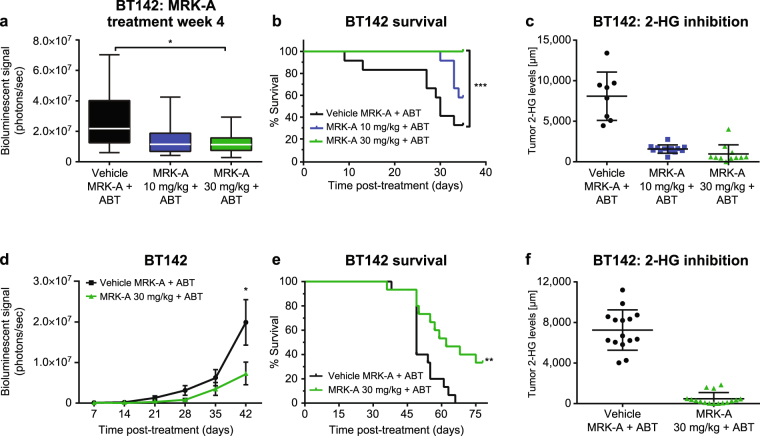
(**a**) Tumor burden as measured by luciferase imaging after 4 weeks of MRK-A dosing in BT142 mouse model. Week 4 luciferase imaging timepoint in BT142 animals treated with either 10 mg/kg or 30 mg/kg MRK-A twice-daily with ABT 50 mg/kg BID co-dosing. (**b**) BT142 mice treated with MRK-A showing a statistically significant survival benefit. Survival curves from vehicle- and MRK-A-treated animals are provided with the associated *p*-value reflecting the median between-group survival estimate (as per the Log-rank Test, Mantel Cox). (**c**) MRK-A inhibits tumor 2-HG levels in treated efficacy study animals. Tumor 2-HG inhibition was measured by LC-MS following 38 days of BID dosing of vehicle, 10 and 30 mg/kg of MRK-A + ABT 50 mg/kg administered orally in the BT142 tumor model. (**d**) Longitudinal bioluminescent imaging of long-term BT142 vehicle and MRK-A treated animals shows extended MRK-A treatment leads to tumor growth inhibition in BT142 mice. (**e**) Long-term administration of MRK-A in BT142 mice confirms survival benefit. Survival curves from vehicle- and MRK-A-treated animals (*p*-value for the between-group differences in median survival estimates using Log-rank Test, Mantel Cox). (**f**) Tumor 2-HG inhibition measured by LC-MS following animal death due to disease-associated morbidity. BT142 animals were dosed orally BID with either vehicle + ABT or MRK-A + ABT. MRK-A significantly reduces 2-HG levels in BT142 tumors. *, **, and *** indicate statistical significance at *P* < 0.05, 0.01, and 0.001 respectively, compared to vehicle control.

Given the ability for MRK-A to potently inhibit 2-HG production in mice bearing intracranially injected tumors and the lack of cell growth inhibition *in vitro*, we sought to understand the potential benefits of 2-HG inhibition in the context of the brain microenvironment in which these lines grow. Large cohorts of animals (N = 12) were examined to enable sufficient statistical power to allow for meaningful interpretation of the study findings given the potential variability associated with injecting small volumes of tumor cells into the striatum of the brain. BT142 animals were dosed BID at 10 and 30 mg/kg with co-dosing of ABT 50 mg/kg over the course of the study. As shown in our prior experiments, these doses should inhibit 2-HG production within the intracranial tumor. We did not observe an immediate dosing benefit in any of the mice treated with MRK-A after 2 weeks of dosing. However, after 4 weeks of treatment, significant tumor growth inhibition was observed in the 30 mg/kg MRK-A group vs vehicle (*P* = 0.02) as measured by bioluminescent imaging (Fig. 3a). Due to the technical limitations of implantation and the temporal and spatial variations observed with bioluminescence imaging, there can be large variations in luciferase signal between animals. This variability can be seen in the tumor burden of imaged vehicle animals (Fig. 3a). In contrast, we found that this inter-animal variation in tumor growth was substantially reduced in the MRK-A treatment groups particularly at the 30 mg/kg dose when compared with vehicle-treated animals (Fig. 3a). These data suggested that prolonged inhibition of tumor 2-HG production substantially inhibited glioma growth intracranially *in vivo*.

#### BT142 MRK-A treatment leads to a survival benefit

Given the confined intracranial context of BT142 tumor expansion, the potential survival benefit associated with MRK-A treatment was closely monitored. Indeed, the tumor growth inhibition observed by imaging in the 30 mg/kg MRK-A treatment group led to a significant survival benefit when compared with the vehicle group (*P* = 0.001). From weeks 3 to 5 of the study, we observed substantial loss of animals in the vehicle group marked by body weight loss and lethargy. After 5 weeks, 70% of the vehicle-treated animals succumbed to disease (Fig. 3b). In contrast, we did not observe similar levels of disease-associated morbidity in the 30 mg/kg MRK-A treatment group (Fig. 3b). In fact, survival analyses between groups could not be performed due to the profound loss of animals in the vehicle group. At the end of the study, we harvested the tumors from all groups to examine the levels of 2-HG inhibition. The mean intratumoral 2-HG measurement was approximately 8 mM for vehicle treated BT142 animals (Fig. 3c). The MRK-A 10 and 30 mg/kg treatment groups showed reductions of 80% and 88%, respectively, in brain tumor 2-HG levels (Fig. 3c). The median survival was not reached in either of the active treatment arms, particularly in the 30 mg/kg group, which had no animal deaths over the 35 day study. This study provided the first evidence that a meaningful preclinical survival benefit could be obtained from prolonged intracranial 2-HG inhibition in IDH1 mutant glioma.

In order to assess the potential long-term benefit associated with MRK-A treatment, an extended study was conducted to assess median overall survival using the BT142 model. Key differences between this experiment and the previous study included the addition of even more animals (N = 18) to each cohort to provide greater statistical power, and earlier initiation of MRK-A dosing at 3.5 weeks (~1 week earlier than the previous study) post injection of BT142 tumor cells. It is important to note that luciferase-based Grade 0–1 animals (Fig. 2a) were enrolled onto this long-term study vs Grade 1 only animals from the initial study. With MRK-A + ABT dosed BID at 30 mg/kg, a tumor growth inhibition trend was observed by bioluminescence beginning at Week 4 of treatment and reaching statistical significance with maximal tumor growth inhibition of 63% vs the vehicle control cohort at Week 6 (Fig. 3d). This finding was further supported by harvesting tumors from Week 4 MRK-A-treated animals for intra-study analysis where they displayed decreased KI-67 immunostaining (Supplementary Fig. S2c, d). After Week 6, animals in the vehicle cohort succumbed to disease-associated morbidity with a median survival of 47 days (Fig. 3e). Again, a meaningful increase in survival was seen in the MRK-A cohort with a 38% increase in median survival vs the vehicle cohort (Fig. 3e). Notably, even after all the vehicle-treated animals had succumbed to disease, over one-third of MRK-A treated animals (7 of 18) survived through Day 78 of the study.

Differences between the two studies in Fig. 3b and e can be attributed to different enrollment criteria (Grade 0–1 vs. Grade 1), study dosing start times, as well as the variable intracranial growth characteristics of BT142. The level of 2-HG inhibition in the MRK-A treatment group was 93% as compared to the vehicle control group (Fig. 3f). To determine if alterations in tumor cell proliferation or apoptosis contributed to the enhanced survival, tumor samples were harvested from MRK-A-treated animals from the aforementioned studies. Given the nature of the survival studies, tumor samples were harvested from moribund animals from both the vehicle and MRK-A treatment groups. From these end-of-study tumors, we did not observe any appreciable differences in cell proliferation (KI-67) or apoptosis (cleaved caspase 3) between these two groups (Supplementary Figs S2a,b; S3c,d). One possible confounding variable in this analysis was the difference in tumor sample harvesting time between the groups, which varied up to several weeks between vehicle- and MRK-A-treated animals due to the differences in survival. Nevertheless, early study KI-67 staining and in life imaging data allowed us to confirm tumor growth inhibition, by MRK-A, in the treated groups over the course of the studies.

Additional follow-up analyses examined possible treatment induced changes in DNA methylation due to the prominent association of IDH1 mutations with this epigenetic phenomenon. Previous work conducted by several groups suggested that reduced 2-HG levels may alter 5-methylcytosine (5-mC) homeostasis in tumor models^7,17^. MRK-A treated BT142 samples were examined for a decrease in tumor 5-mC levels, which would be consistent with a reduction in DNA methylation. Compared to vehicle treated animals, a statistically significant reduction in 5-methylcytosine levels were observed in MRK-A 30 mg/kg tumor sections (*P* = 0.0004) versus vehicle control tumors, as measured by immunohistochemistry (Supplementary Fig. S2e,f). Since the reduced 5-mC may also lead to changes in 5-hydroxymethylcytosine (5-hmC) levels, we also looked for increased levels of 5-hmC in MRK-A-treated tumors. However, only a non-significant increase in 5-hmC levels was seen in the MRK-A treated samples (Supplementary Fig. S3a,b). The observed changes in DNA methylation patterns may indicate gene expression in the tumor is ultimately modified. These data suggested that IDH1 mutant inhibitor treatment may reverse the DNA hypermethylation phenotype associated with IDH1 mutant glioma.

### MRK-A displays mutant-selective efficacy

#### IDH1 wildtype U87MG GBM glioma model displays no tumor growth inhibition benefit with MRK-A

**Figure 4:**
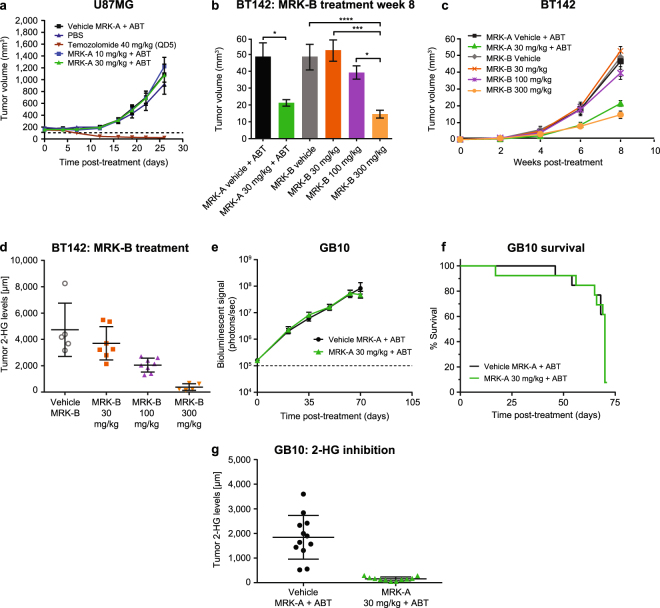
(**a**) MRK-A treatment does not inhibit tumor growth in wildtype IDH1 U87MG tumor xenografts. U87MG animals were dosed BID with either 10 or 30 mg/kg MRK-A + ABT. Temozolomide positive control animals were treated QD for the first 5 days of the study. Tumor volumes were measured twice-weekly by caliper measurement. (**b**) 8 weeks of treatment with MRK-A and MRK-B induces tumor growth inhibition in BT142 animals. BT142 tumor volumes were measured by MR imaging after 8 weeks of treatment with either MRK-A + ABT or MRK-B. Compounds were dosed orally BID at the doses labeled. (**c**) Longitudinal monitoring of BT142 tumor growth using MRI. 8 weeks of MRK-A + ABT and MRK-B treatment induces tumor growth inhibition. Treatment started 3 weeks post implantation. (**d**) MRK-B significantly reduces 2-HG levels in BT142 intracranial tumors. Tumor 2-HG inhibition was measured by LC-MS postmortem. BT142 animals were dosed orally twice-daily with MRK-B at the stated doses. (**e**) Longitudinal bioluminescent imaging of vehicle- and MRK-A-treated GB10 animals demonstrates GB10 tumor model does not respond to MRK-A treatment. (**f**) GB10 survival curves for vehicle- and MRK-A-treated animals. (**g**) MRK-A significantly reduces 2-HG levels in GB10 tumors. Tumor 2-HG inhibition was measured by LC-MS postmortem. GB10 animals were dosed orally twice-daily with either vehicle + ABT or MRK-A + ABT. *, ***, and **** indicate statistical significance at *P* < 0.05, 0.001, and 0.0001 respectively.

The wildtype IDH1 U87MG human glioma flank xenograft model was used to determine if MRK-A treatment effects were IDH1 mutant-specific. Mice were treated with MRK-A at 30 mg/kg twice-daily to mimic the dosing used in prior studies. As expected with a mutant IDH1-selective compound, no tumor growth inhibition was observed following MRK-A treatment in this IDH1 wildtype tumor model (Fig. 4a). U87MG is known to be sensitive to temozolomide (TMZ), so it was used as a positive control. Treatment of U87MG animals with TMZ elicited robust anti-tumor efficacy in this model (Fig. 4a). Since U87MG does not produce appreciable levels of 2-HG, no measurements of 2-HG inhibition were performed in this experiment. These results suggest that MRK-A inhibition of 2-HG production elicited mutant-specific anti-tumor efficacy only in the BT142 glioma model.

#### MRK-B displays better tumor growth inhibition

Given the potential therapeutic value of 2-HG inhibition in glioma, we sought to improve the properties of our mutant-selective inhibitors. MRK-B was discovered from the same chemical series as retaining high brain penetrance without the need for ABT co-dosing to boost exposure. In preclinical experiments, this compound was found to be well tolerated at doses up to 300 mg/kg when administered orally BID. Both MRK-A and MRK-B were tested head-to-head in a BT142 efficacy study, using MRK-A as a benchmark for efficacy. Over the course of the study, increasing tumor growth inhibition was observed with MRK-B treatment across the three escalating dosing groups of 30, 100, and 300 mg/kg, respectively (Fig. 4b). At 300 mg/kg, the highest dose tested, MRK-B displayed substantial tumor growth inhibition compared with the vehicle control group (*P* ≤ 0.0001). Impressively, MRK-B at this dose was able to show statistically significant tumor growth inhibition versus the MRK-A 30 mg/kg + ABT BID group at Week 8 (*P* = 0.02, Fig. 4c). The vehicle + ABT group was indistinguishable from the vehicle alone group (i.e. without ABT, *P* = 0.99), demonstrating that the observed anti-tumor efficacy was mediated by our IDH1 mutant-selective compounds rather than ABT. At the end of the study, we examined 2-HG inhibition across the different MRK-B dosing groups. Tumor 2-HG production was inhibited by 22%, 57%, and 93% for the 30, 100, and 300 mg/kg dose groups, respectively (Fig. 4d). These results highlight the potential for improved compound characteristics to increase the therapeutic potential of IDH1 mutant inhibitors in the IDH1 mutant glioma setting.

#### MRK-A provides no efficacy benefit in the GB10 glioma model

The GB10 model, a novel IDH1 R132H mutant glioma intracranial model, was developed to test if the observed BT142 survival benefit could be seen in other mutant glioma models. As mentioned, IDH1 mutant glioma models are extremely difficult to propagate *in vivo*. GB10 model development required well over a year of serial *in vivo* propagation to achieve the following work. This model was derived from an anaplastic astrocytoma obtained from the right temporal lobe of a 28-year-old female. The tumor sample was sequenced and confirmed to harbor both a heterozygous IDH1 R132H mutation and a homozygous TP53 R273C canonical hotspot mutation. Additional analysis found GB10 to be wildtype for PTEN, CIC, FUBP1, IDH2, and PIK3CA exons 9 &20 (Supplementary Table S1). As performed in the BT142 model, tumor cells were modified to express luciferase and injected into the striatum of immune-deficient mice. In contrast to BT142, GB10 was found to grow as a solid tumor mass and showed a substantially longer *in vivo* median survival time of 17 weeks. Typical *in vivo* tumor 2-HG levels in this model were measured to be approximately 2 mM, which is substantially lower than that observed for BT142. As previously shown in Fig. 2f, MRK-A robustly inhibited GB10 2-HG production at all doses tested above 3 mg/kg. The development of the GB10 model enabled us to better understand the heterogeneity in the treatment response that may be observed clinically for IDH1 mutant glioma.

An efficacy study was performed using GB10 to assess intracranial IDH1 mutant glioma growth inhibition following MRK-A treatment. As per previous experiments, the 30 mg/kg BID dose was administered and *in vivo* bioluminescent imaging was used to monitor tumor progression. Following 10 weeks of dosing with MRK-A, no tumor growth inhibition (Fig. 4e) or survival benefit (Fig. 4f) was observed in the GB10 cohort. Due to the lack of observed efficacy in this study, both vehicle- and MRK-A-treated animals were euthanized and tumor samples were harvested for follow up analyses. As observed previously, MRK-A achieved near complete inhibition of tumor 2-HG production relative to control animals (Fig. 4g). The lack of observed efficacy in GB10 following MRK-A treatment was a curious finding. However, recent human clinical data in IDH1 and IDH2 mutant AML, where not all mutant patients respond to inhibitor treatment, points to the plausibility that not all mutant glioma patients will respond to such therapy.

### Gene expression differences between BT142 and GB10

**Figure 5:**
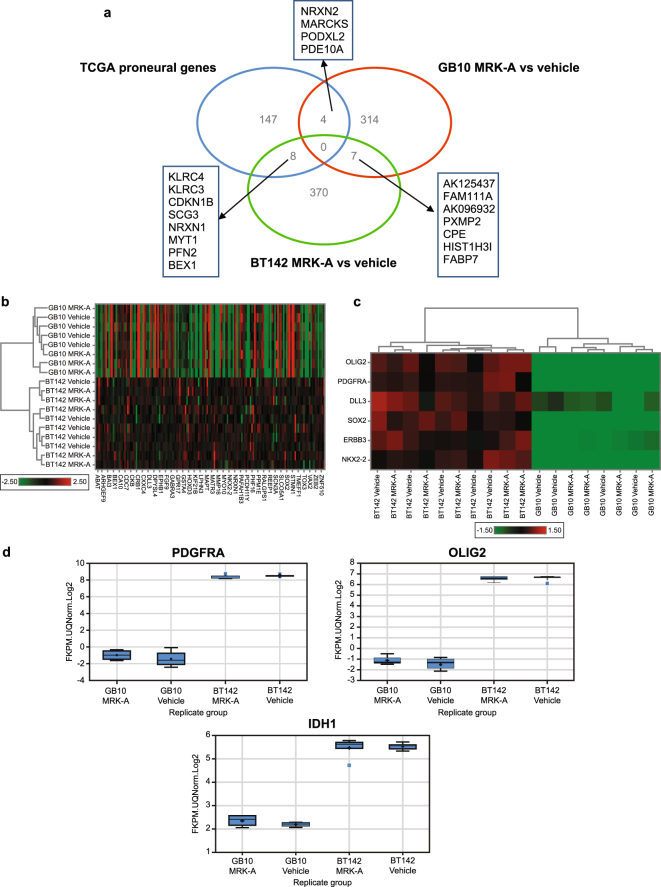
(**a**) Differentially expressed genes between BT142, GB10 and TCGA GBM datasets. A Venn diagram showing overlap between gene expression patterns for BT142 (green), GB10 (red) and the TCGA proneural geneset (blue) from Verhaak *et al*.^5^. For BT142 and GB10 tumors, differentially expressed genes were compared in MRK-A-treated animals relative to vehicle-treated animals. All animals were dosed BID; MRK-A was dosed at 30 mg/kg and vehicle with ABT was dosed at 50 mg/kg. Genes were considered differentially expressed if *p*-values were < 0.01 for MRK-A versus vehicle groups. (**b**) Hierarchical clustering using all proneural genes from Verhaak *et al*. to compare BT142 to GB10. Heat map showing differential expression of the TCGA proneural geneset in IDH1 mutant models^5^. (**c**) Heat map showing differential expression of key proneural genes in BT142 and GB10. Heat map showing differential expression of key TCGA proneural genes highlighted by Verhaak *et al*., between BT142 and GB10 treated and vehicle treated tumors^5^. (**d**) Individual proneural associated genes display large differences in expression between BT142 and GB10. RNA-Seq data for differential expression of PDGFRA, OLIG2 and IDH1, respectively between BT142 and GB10 treated and vehicle tumors.

In an effort to understand the dramatic differences in therapeutic response to IDH1 inhibitors between BT142 and GB10, we conducted RNA sequencing to compare gene expression profiles between these two models. We searched for tumor gene expression changes between the two models following treatment with MRK-A *in vivo*. For each model, we observed over 300 genes that were significantly altered (*P* < 0.01) in response to drug treatment (Supplementary Table S2a,b). However, only 7 genes were shared amongst each respective model (Fig. 5a). From this limited gene list, carboxypeptidase E (CPE), fatty acid binding protein 7 (FABP7) and peroxisomal membrane protein 2 (PXMP2) were all previously found to be associated with human cancer. Upon comparing these two models, informatic analysis revealed that GB10 was generally transcriptionally silent in response to MRK-A treatment. In fact, if a 1.5-fold expression change cutoff was imposed on the GB10 dataset, no genes were found to be upregulated. This contrasts with the 245 genes with marked increase in the BT142 dataset. Additional details on the informatics analysis can be found in the methods section. Thus, the transcriptionally quiet nature of MRK-A-treated GB10 tumors may explain differences in the observed efficacy between these models.

Given that IDH1 mutant patients cluster into the proneural molecular subgroup for glioma^5^, we examined the differential expression of genes comprising this molecular subgroup in the BT142 and GB10 datasets. A pronounced trend towards transcriptional downregulation of proneural genes was observed in GB10 compared with the BT142 model (Fig. 5b). Both the vehicle and MRK-A treated GB10 groups displayed strong downregulation of proneural genes relative to BT142 (Fig. 5b). This trend was most apparent for key genes known to be associated with the proneural subgroup, as demonstrated by the nearly complete lack of expression of *OLIG2*, *PDGFRA*, *ERBB3*, *DLL3*, *NKX2.2* and *SOX2* genes (Fig. 5c). For example, the *PDGFRA* gene is known to be mutated in the proneural subtype of glioma, its expression was elevated ~16-fold in BT142 as compared with GB10 (Fig. 5d). Furthermore, the oligodendrocyte marker and key component of the proneural signature, *OLIG2*, was also highly differentially expressed in BT142 vs GB10 (Fig. 5d). Since IDH1 mutations are prominently found in the proneural subtype of glioma, these findings suggest that the BT142 model may be most similar to classical IDH1 mutant patients^5^. Interestingly, IDH1 mRNA levels were found to be lower in the GB10 model which also may explain the lower levels of tumor 2-HG *in vivo* (Fig. 5d). To confirm this at the protein level, western blot analysis for mutant IDH1 R132H was conducted on GB10, BT142, MOG-R132H (positive control), and as a negative control the HT1080 IDH1 R132C line (Supplementary Fig. S4a–d). Relative to BT142 and MOG-R132H, GB10 displays substantially reduced levels of mutant IDH1 R132H protein. Given the reduction in proneural gene signature, IDH1 RNA expression and IDH1 R132H protein levels, in conjunction with the observed relative 2-HG decrease in GB10 tumors, these findings may explain the lack of observed efficacy with MRK-A treatment in this model. These results suggest that GB10 may no longer be dependent on the IDH1 mutation and thus 2-HG production.

## Discussion

This report describes the discovery of brain-penetrant IDH1 mutant-selective small molecule inhibitors capable of potently inhibiting 2-HG production in patient-derived orthotopic IDH1 mutant glioma xenograft models. Much like other targeted therapies for genetically defined patient populations, IDH1 mutant gliomas appear to be selectively sensitive to 2-HG inhibition in order to elicit an anti-tumor response. For both the IDH1 R132H mutant BT142 and GB10 models described herein, MRK-A was able to achieve robust intracranial 2-HG inhibition in the orthotopic mouse brain tumor setting. Yet, only BT142 displayed significant tumor growth inhibition resulting in a measurable survival benefit. This contrasted with GB10, where no such benefit was observed. Pronounced differences in the gene expression patterns between BT142 and GB10 tumors were observed following MKR-A treatment. Similar to other mutant selective inhibitors, these findings suggest that there may be heterogeneity in the response to IDH1 mutant inhibitors when used clinically in glioma patients.

IDH1 mutations are known to be associated with distinct gene expression changes across several tumor types^5,8,21,22^. It is plausible that tumor gene expression changes may occur due to inhibition of 2-HG production given the influence of this metabolite on the epigenetic state of tumors^7,9,22^. As such, reversal of the 2-HG phenotype should then involve changes to gene expression as seen for BT142 treatment with MRK-A. While several statistically significant gene expression alterations were observed in BT142 tumors, very few changes were seen in GB10 tumors nor was this model responsive to MRK-A treatment. Thus, further characterization of additional models, both inhibitor responsive and non-responsive, is needed to understand the potential predictive value of preclinical modeling in IDH1 mutant patient-derived glioma models.

It has recently been shown that elevated levels of 2-HG impairs the function of alpha-ketoglutarate-dependent dioxygenases involved in the regulation of DNA and histone methylation^7^. It is conceivable that tumor growth inhibition may occur in those tumors that alter their gene expression in response to the removal of 2-HG. In MRK-A-treated BT142 tumors, we observed broad upregulation of gene expression which was consistent with decreased levels of 5-methylcytosine and the reversal of DNA hypermethylation. These findings are similar to the results seen in previous studies involving the inhibition of 2-HG production by mutant IDH1^7,21,22^. These findings are also consistent with previous work employing inhibitors to reduce tumor 2-HG levels preclinically in a subcutaneous IDH1 mutant glioma model^17^. Interestingly, we observed that the tumor suppressor CDKN1A/p27 was upregulated in response to MRK-A treatment in BT142. The function of p27, as a potent cell cycle inhibitor, is well documented and suggests that the observed tumor growth inhibition in BT142 may be due at least in part to elevated p27 expression. However, given the substantial number of gene expression alterations observed with MRK-A treatment in BT142, more in depth analysis needs to be conducted.

In comparison to BT142, GB10 displayed far fewer gene expression changes even with near complete 2-HG inhibition by MRK-A treatment. Due to the lack of gene expression changes in this model, we were not able to develop a causal explanation for the lack of efficacy with MRK-A. Nevertheless, this model was found to produce far lower levels of intra-tumor 2-HG than BT142. This observation coupled with the finding that BT142 expresses 4-fold higher levels of mutant IDH1 than GB10 suggests that this latter model may no longer require 2-HG for maintaining its tumorigenic state. It is intriguing to speculate that stronger expression of IDH1 R132H, as seen in BT142, may predict tumor response in low grade glioma patients, but this hypothesis requires additional preclinical and/or clinical validation. Additionally, we found that *PDGFRA* and other key proneural genes are expressed at lower levels in GB10 compared with BT142. Indeed, the observed low expression of a large constituent of the proneural gene set in GB10 suggests that this tumor type may be quite different than most IDH1 mutant patient tumors. Given these differences and the fact that the TCGA proneural subgroup encompasses IDH1 mutant glioma, GB10 may simply be an outlier that is not representative of the general IDH1 mutant glioma population. Though limited by the number of orthotopic IDH1 mutant models available, these data suggest that the ability to inhibit 2-HG over a sustained period of time may provide a meaningful survival benefit in patients with IDH1 mutant glioma.

The discovery of IDH1 mutations in human glioma and the recent clinical findings in IDH1- and IDH2-mutant acute myeloid leukemia patients highlights the potential clinical utility of brain-penetrant IDH1 mutant-selective compounds. The results from the BT142 IDH1 mutant tumor model reported herein, which shows similarity with the proneural subset of glioma patients from TCGA, represents an enticing potential opportunity for the application of a brain-penetrant IDH1 mutant inhibitor that may benefit patients clinically.

## Methods

### Cell Line Culturing Conditions

HT1080 cell line was cultured in DMEM GlutaMax (Gibco, Grand Island, NY, 10566) with 10% heat-inactivated fetal bovine serum (Sigma, St. Louis, MO, F4135). MOG-G-UVW was cultured in DMEM/F12 GlutaMax (Gibco, Grand Island, 10565) with 10% heat-inactivated fetal bovine serum (Sigma, St. Louis, MO, F4135) and 5 µg/mL blasticidin (Thermofisher, Grand Island, NY, A1113902). BT142 and GB10 glioma cell lines were maintained in Neuro Cult XF Basal Medium with Supplement (StemCell Technologies, Vancouver, BC, Canada, 05761), 20 ng/mL rhEGF (PeproTech, Rocky Hill, NJ, 100–15), 100 ng/mL PDGF (PeproTech, Rocky Hill, NJ, 100–13 A), 20 ng/mL recombinant human bFGF (Invitrogen, Grand Island, NY, 13256), 2 µg/mL Heparin (Stemcell Technologies, Vancouver, BC, Canada, 07980), and Penicillin-Streptomycin (Gibco, Grand Island, NY, 15140).

### qPCR

FAM-labeled TaqMan Primers for Nanog, Oct-4, Sox-2, GFAP, GALC, CNP and Nestin were obtained from Applied Biosystems Inc. (catalog number 4331182). Quantitative RT-PCR experiments were performed on Applied Biosystems QuantStudio 7 Flex Real-Time PCR System. Samples were run in triplicate, normalized to GAPDH for loading control, and expressed as ratios to non-treated samples.

### SDS-PAGE and western blotting

Cell pellets (5–10 × 10^6^) were lysed with RIPA buffer and sonicated. 20 µg of protein was run on SDS-PAGE and transferred to nitrocellulose. Blots were blocked in Odyssey blocking buffer (Li-COR, 927–40000) for 1 hour, and incubated overnight at 4 °C in primary antibody (IDH1_R132H_, Dianova, 1:500; IDH1_wt_, Cell Signaling Technologies, 1:1000; Beta actin, Cell Signaling Technologies, 1:1000). Blots were washed, then incubated for 1 h with labeled secondary antibody (anti-mouse or anti-rabbit IRDye, Li-COR, 1:5000). Blots were washed and bands were visualized on the Odyssey CLx.

### Generation of lentiviral constructs

The luciferase Luc-2 fragment was subcloned into pFUGW vector backbone using EcoRI/BamHI. The pFUGW plasmid is available through Addgene (Cambridge, MA).

### Lentiviral production, infection and cell line generation

Lentivirus was produced by transient transfection in human embryonic kidney (HEK) 293 T cells. Cells were obtained from the ATCC (ATCC, Manassas, VA #CRL-11268). Transfections were carried out according to manufacturer’s instructions using Fugene6 (Roche). ~1 × 10^8^ BT142 and GB10 cells were infected with concentrated virus (0.5–1 × 10^9^ TU/ml). 1 × 10^5^ cells were lysed 48 h after infection and firefly and renilla luciferase were measured on a GloMax96 Microplate Luminometer using the Dual Luciferase Assay System (Promega, Madison, WI).

### Cell proliferation Assay

Cell proliferation studies were conducted in triplicate using 96-well plates with a 72 h proliferation schedule. Cell viability was assessed by using CellTiter-Glo Luminescent Cell Viability Assay (Promega, Madison, WI, G7570).

### 2-HG measurements

An ABI API4000 mass-spectrometer equipped with turbo-ion spray interface was used for measurements. The mass spectrometer was operated in negative ion mode with high resolution for Q1 and Q3.The following single reaction-monitoring transitions were monitored: 146.782/128.812 for 2-hydroxyglutarate and 116.954/72.957 for methylmalonic acid used as internal standard.

The brain tissue harvested for 2-HG measurements used ipsilateral sections including the injection site and tissue in its immediate vicinity (several millimeters around the injection site). Corresponding contralateral hemisphere sections were collected as well, and both ipso- and contralateral sections are displayed in black triangles (Fig. 2d). A liquid-liquid extraction procedure was used for biological samples. A sample volume of 50 µL was acidified by adding 5 µL of 5% formic acid in H_2_O and extracted with 1 mL of MTBE. The organic top layer was then evaporated and reconstituted in 100 µL of internal standard (IS, methylmalonic acid) in 20 mM CH_3_COONH_4_ in H_2_O. The extracted samples were analyzed on a Chromolith Performance RP-18e (100–3 mm) column using 0.2% formic acid in water (mobile phase A) and 0.2% formic acid in methanol (mobile phase B). A 1.0 minute gradient was utilized going from 50% to 90% of mobile phase B for a total run time of 3.0 minutes.

### *In vivo* xenograft models

Thirty thousand (BT142) and one hundred thousand (GB10) cells in 3 µL of culture medium were implanted into the right striatum of 6–8 week-old CB-17 SCID mice (Charles River Laboratories, Wilmington, MA) using a small animal stereotaxic instrument with digital display (KOPF, Tujunga, CA). Tumor was localized to the striatum using consistent coordinates.

Previously implanted mice were monitored by Magnetic Resonance or Bioluminescence imaging for brain tumor formation. All animal procedures were approved by the Institutional Animal Care and Use Committee and carried out in accordance to approved protocols. All experimental protocols were approved by the Institutional Animal Care and Use Committee.

Morphological changes observed in the dissected brain revealed that tumor growth was primarily in the right hemisphere, the site of implantation. Mice displaying distress and body weight loss as defined by the Institutional Animal Care and Use Committee protocol were sacrificed and the animals’ brains were removed for 2-HG sampling or propagation. BT142 and GB10 cells were propagated *in vivo* through tissue dissociation from mouse brains using a Neural Tissue Dissociation Kit (P) 13-092-628 and processed on a GentleMACS Dissociator (Miltenyi Biotech, San Diego, CA). Viable cells were counted on a Vi-CELL Viability Analyzer (Beckman Coulter Life Sciences, Indianapolis, IN) using trypan blue exclusion. Viable cells were cultured 3–4 days in Neuro Cult XF Medium (StemCell Technologies, Vancouver, BC, Canada) supplemented with EGF (20 ng/mL; Peprotech, Rocky Hill, NJ), rhFGF at 20 ng/mL (R&D Systems Minneapolis, MN), Heparan sulfate at 2 µg/mL (Sigma, St. Louis, MO), and 1% Penicillin-Streptomycin (Gibco, Grand Island, NY) as sphere cultures incubated at 37 °C with 5% CO_2_.

GB10 genetic mutation analysis was conducted in IDH1, IDH2, TP53, PTEN, FUBP1, CIC and PIK3CA using Sanger sequencing against all coding exons unless otherwise noted. Brain tumor formation, growth, and kinetics were monitored by MRI or Bioluminescence for all *in vivo* studies. In all treatment studies, the dosing interval was BID. Treatment starting point was based on tumor burden, which was determined by MRI volumetric readout or bioluminescent signal.

### Bioluminescence imaging

Bioluminescence imaging was performed as described by Contag *et al*.^23^. Briefly, D-Luciferin Potassium (Perkin Elmer, Waltham, MA) was dissolved in PBS at 30 mg/ml. Mice were injected subcutaneously with a dose of 150 mg/kg while anesthetized using 2–3% isoflurane in 2 L/min of oxygen. Mice were imaged 15 minutes post D-Luciferin injection under anesthesia using an IVIS Spectrum system (Perkin Elmer, Waltham, MA). The field of view was set to 25.6 cm, at medium sensitivity, and integration times ranged from 1 second to 2 minutes depending on signal intensity. Images were analyzed using Living Image 4.4 software (Perkin Elmer, Waltham, MA). Regions of interest were manually selected, and the output signal intensity, total flux, was expressed as photons per second.

### Magnetic Resonance Imaging

Whole brain, anatomical MRI was performed using a 7 T Bruker BioSpec 70/30 USR and Paravision software (PV5.0, Bruker Biospin, Billerica, MA). Mice were anesthetized with a continuous flow of 2% isoflurane in 2 L/min of oxygen and maintained as such throughout the scanning procedure. Respiratory monitoring was performed during the acquisition using a pneumatic sensor (Model 1030, SA Instruments, Inc., Stony Brook, NY). Following whole brain coronal scout image acquisition to improve positioning, a multislice T2-weighted rapid acquisition with relaxation enhancement (RARE) sequence was acquired. The matrix size for each image was 128 × 96, the field of view was 20 × 15 mm, the TE/TR = 15/3000 ms, NA = 4, RARE factor = 8, and the flip angle of the excitation pulse was 180°. Total preparation and MR scan time, including anesthetizing the mice, was approximately 7 minutes. The raw data were converted to Analyze format using MATLAB (MathWorks, Inc., Natick, MA) and analyzed using Amira® 5.4.2 image analysis software (Mercury Computer Systems, Inc., Chelmsford, MA). Data was analyzed by visually identifying tumor in MRIcro and graded based on a scale of 0–5; for quantitative analysis, Amira® 5.4.2 image analysis software was used and the entire tumor was manually selected based on T2 hyperintensity of the tumor.

### Immunohistochemistry

Formalin-fixed paraffin-embedded orthotopic human tumor xenograft containing mouse brains were sectioned at 5 μm thickness and analyzed for Ki-67 (Thermo Scientific, Grand Island, NY), pHH3 (Epitomics, Burlingame, CA), CC3 (Cell Signaling Technologies, Danvers, MA), 5-mC (EMD Millipore, Billerica, MA), Nestin (Spring Biosciences, Cat#M4030) and IDH1 R132H (Dianova, Hamburg, Germany). Automated staining was done using the ChromoMap Kit on the Discovery XT (Ventana Medical Systems, Tucson, AZ) under standard conditions.

### Gene expression analysis

RNA sequencing data were generated from the Illumina RNA sequencing platform (Illumina Inc., San Diego, CA). Expression analysis was conducted using the Omicsoft analysis tool (QIAGEN, Cary, NC). 1 μg of total RNA was used to prepare libraries using the TruSeq Stranded Total RNA RiboZero protocol for sequencing on the Illumina HiSeq. A read length of 50 paired end reads with 3 GB of total coverage was generated. Data was analyzed using the OmicSoft QC and FPKM pipeline. Data was normalized to the number of reads (FKPM), Upper Quartile normalized and Log2 transformed. Differential gene expression was determined using the DESeq. 2 function in OmicSoft using a p-value threshold of 0.01. Boxplots were generated in OmicSoft using the Boxplot_Rstyle function which displays a box bounded by the 25% and 75% ranked sample, with a line indicating the median, and the “whiskers” indicating the bounds of 1.5*IQR (Inter-quartile range). Only values outside the whiskers are plotted.

A Volcano plot and Principal Component Analysis (PCA) for GB10 and BT142 vs. vehicle (Supplementary Fig. S5a–c) as well as all gene expression differences (Supplementary Table S2a,b) are detailed in the supporting information.

### Statistical analysis

All data was analyzed in GraphPad Prism 6.04 software (GraphPad, La Jolla, CA). Statistical analysis was performed using log rank (Mantel-Cox) test (Figs 1f, S2c,). Figure 3a,b,d, and e used unpaired t-test. Figure 4c used two-way (time x group) repeated measures ANOVA. Statistical significance was established for *p* < 0.05.

### High throughput screening to identify inhibitors of IDH1 R132H

We conducted a high throughput screen of the Merck screening collection (~2 million compounds) using a coupled assay system to identify inhibitors of the IDH1 enzyme with an R132H mutation; NOG (*N*-Oxalylglycine) was used as a control compound. All hits were subsequently counter-screened against the wild-type enzyme and only compounds which selectively inhibited the mutant enzyme were advanced further. After chemical validation, representative compounds were studied in the cellular assays (such as MOG or HT1080 assays described in this manuscript) where we found several compound clusters that weakly inhibited the production of 2-HG. Focusing on our goal to find an *in vivo* active tool we selected a single chemical series as our most promising starting point which was further optimized for potency and properties to arrive at MRK-A. Further details on the outcome of our screen and the medicinal chemistry optimization towards MRK-A as well as the identification of MRK-B will be published elsewhere in due course.

### Coupled assay system

Alpha ketogluterate (aKG) reacts with R132H IDH1 to form 2-hydroxygluterate and NADP+. The NADP+ produced reacts with glyceraldehyde phosphodehydrogenase (GAPDH) along with added glyceraldehyde phosphate (GAP) to form 1,3 phosphoglyceraldehyde (1,3PGA). 1,3 PGA is utilized as a substrate by 3- phosphoglyceraldehyde kinase (PGK) together with added ADP to form ATP, which is quantified by luciferase luminescence (Vialight) readout.

### Assay protocol

Assay buffer containing R132H IDH1 enzyme and coupled assay components was added to white 384 well assay plates containing test compounds plus 2 control compounds. Wells containing DMSO received assay buffer with or without enzyme to generate high and low activity responses for data analysis. Following 30 minutes pre-incubation the reaction was initiated by addition of aKG substrate. The reaction was run at room temperature for 1 hour and stopped by addition of an equal volume of kinase-GLO detection reagent. Activity was captured with an Envision Spectrophotometer equipped with a luminescence reader.

### Determination of MRK-A brain penetration in naïve C57Bl6 mice

Total brain levels, expressed as brain/blood ratio (Kp,_brain_) were measured by AUC_brain_/AUC_blood_ in mice after oral administration. Free fraction of MRK-A in biological matrix was determined by *in vitro* blood and brain binding assay. Kp_uu, brain_ was calculated by the following equation:

$${{\rm{Kp}}}_{{\rm{uu}},{\rm{brain}}}={{\rm{AUC}}}_{{\rm{brain}}}{/\text{AUC}}_{{\rm{blood}}}\times ({\text{fu},}_{{\rm{brain}}}{/\text{fu},}_{{\rm{blood}}})$$

*In vitro* blood and brain binding assay was carried out on a HT-Dialysis plate.

Unbound fraction (fu) of MRK-A in the brain homogenate and diluted blood were calculated by the ratio of the buffer side response to the brain homogenate/blood side response, and unbound fraction (fu,blood and fu,brain) of MRK-A in non-diluted blood and tissue were calculated from measured fu in homogenate and diluted blood with the following equation:

$${\rm{fu}}=(1/{\rm{D}})/[(1/\text{fu}-1)+1/{\rm{D}})].\,\,({\rm{D}}\,{\rm{is}}\,{\rm{dilution}}\,{\rm{factor}}).$$

An oral absorption model was used as the *in vivo* screening model to identify brain penetration of MRK-A. Female naïve C57Bl6 mice (n = 5) were orally dosed with MRK-A at 30 mg/kg in PEG300/50% Captisol/H_2_O (30/50/20) (with ABT 50mpk PO @ 5 mL/kg 2 h prior to MRK-A dosing). At 1, 2, 6 and 12 hour post-dose blood samples (>10 μL/time point) were collected via tail clip, and then immediately diluted with 3-fold volume of 0.1 M sodium citrate. Brain tissue was harvested at the 12 hour time point and homogenized in 3x volume of water. All samples were stored at ~−20 °C prior to LC/MS/MS analysis. At trough, the 12 hour time point, the average unbound brain to plasma ratio was determined to be 1.04 (Supplementary Fig. S6), which is indicative of good passive brain penetration.

## Electronic supplementary material

Supplementary information

## References

[1] Balss J (2008). Analysis of the IDH1 codon 132 mutation in brain tumors. Acta Neuropathol..

[2] Cairns RA, Mak TW (2013). Oncogenic isocitrate dehydrogenase mutations: mechanisms, models, and clinical opportunities. Cancer Discov..

[3] Parsons DW (2008). An integrated genomic analysis of human glioblastoma multiforme. Science.

[4] Yan H (2009). IDH1 and IDH2 mutations in gliomas. N. Engl. J. Med..

[5] Verhaak RG (2010). Integrated genomic analysis identifies clinically relevant subtypes of glioblastoma characterized by abnormalities in PDGFRA, IDH1, EGFR, and NF1. Cancer Cell.

[6] Dang L (2009). Cancer-associated IDH1 mutations produce 2-hydroxyglutarate. Nature.

[7] Xu W (2011). Oncometabolite 2-hydroxyglutarate is a competitive inhibitor of alpha-ketoglutarate-dependent dioxygenases. Cancer Cell.

[8] Guilhamon P (2013). Meta-analysis of IDH-mutant cancers identifies EBF1 as an interaction partner for TET2. Nat. Commun..

[9] Noushmehr H (2010). Identification of a CpG island methylator phenotype that defines a distinct subgroup of glioma. Cancer Cell.

[10] Cooper LA (2010). The proneural molecular signature is enriched in oligodendrogliomas and predicts improved survival among diffuse gliomas. PLoS One.

[11] Luchman HA (2012). An *in vivo* patient-derived model of endogenous IDH1-mutant glioma. Neuro. Oncol..

[12] Hirata M (2015). Mutant IDH is sufficient to initiate enchondromatosis in mice. Proc. Natl. Acad. Sci. USA.

[13] Saha SK (2014). Mutant IDH inhibits HNF-4alpha to block hepatocyte differentiation and promote biliary cancer. Nature.

[14] Sasaki M (2012). IDH1(R132H) mutation increases murine haematopoietic progenitors and alters epigenetics. Nature.

[15] Wang F (2013). Targeted inhibition of mutant IDH2 in leukemia cells induces cellular differentiation. Science.

[16] Capper D, Zentgraf H, Balss J, Hartmann C, von Deimling A (2009). Monoclonal antibody specific for IDH1 R132H mutation. Acta Neuropathol..

[17] Rohle D (2013). An inhibitor of mutant IDH1 delays growth and promotes differentiation of glioma cells. Science.

[18] Guo Y (2011). Expression profile of embryonic stem cell-associated genes Oct4, Sox2 and Nanog in human gliomas. Histopathology.

[19] Perez CA, Aguilar-Morante D, Morales-Garcia JA, Dorado J (2008). Cancer stem cells and brain tumors. Clin. Transl. Oncol..

[20] Singh SK, Clarke ID, Hide T, Dirks PB (2004). Cancer stem cells in nervous system tumors. Oncogene.

[21] Figueroa ME (2010). Leukemic IDH1 and IDH2 mutations result in a hypermethylation phenotype, disrupt TET2 function, and impair hematopoietic differentiation. Cancer Cell.

[22] Turcan S (2012). IDH1 mutation is sufficient to establish the glioma hypermethylator phenotype. Nature.

[23] Contag CH (1997). Visualizing gene expression in living mammals using a bioluminescent reporter. Photochem. Photobiol..

