# Supplementary material for: A Brain Penetrant Mutant IDH1 Inhibitor Provides In Vivo Survival Benefit
Source: Sci Rep. 2017 Oct 23;7:13853. doi: 10.1038/s41598-017-14065-w (PMC5653818; doi:10.1038/s41598-017-14065-w)
Supplement: Supplementary file 1 — Supplementary information [file 41598_2017_14065_MOESM1_ESM.doc]

**Supplementary**

**A Brain Penetrant Mutant IDH1 Inhibitor Provides *In Vivo* Survival Benefit**

Johnny Kopinja, Raquel S. Sevilla, Diane Levitan, David Dai, Amy Vanko, Edward Spooner, Chris Ware, Robert Forget, Kun Hu, Astrid Kral, Peter Spacciapoli, Richard Kennan, Lata Jayaraman, Vincenzo Pucci, Samanthi Perera, Weisheng Zhang, Christian Fischer, and Michael H. Lam

**Supplementary Figure Legends**

**Supplementary Figure S1.** (**a**) HT1080 and (**b**) GB10 cell viability to MRK-A treatment. (**c**) MRK-A in vitro dose response with BT142 IDH1 R132H glioma cells over 1 week. (**d-f**) MRK-A treated BT142 qPCR expression analysis for glial markers GFAP, GALC, and CNP.

**Supplementary Figure S2.** (**a**) KI-67 cell proliferation analysis in BT142 tumor samples from the long-term MRK-A study. BT142 tumors no longer display decreased proliferation by the end of study. (**b**) Apoptosis (cleaved caspase 3) was also unchanged in these samples. (**c-d**) 4 Week MRK-A treatment intra-study analysis reveals a decrease in tumor cell proliferation. Proliferation was measured by Ki-67 immunohistochemistry staining on paraffin-embedded tumor sections. (**c**) Graph represents quantification of positive staining for Ki-67. (**d**) Representative images of Ki-67 staining in vehicle- and MRK-A-treated tumors. (**e-f**) Immunohistochemistry for 5-methycytosine levels shows a significant decrease in 4-week MRK-A treated intra-study tumor sections versus vehicle control tumors * indicates statistical significance between MRK-A 30 mg/kg +ABT and Vehicle MRK-A + ABT at *P* < 0.05 , *** indicates statistical significance between MRK-A 30 mg/kg and Vehicle MRK-A + ABT at *P*< 0.001.

**Supplementary Figure S3.** (**a-b**) MRK-A treatment leads to a minor increase in 5-hydroxymethycytosine levels in BT142 4-week MRK-A treated intra-study tumors. (**c-d**) 4-week MRK-A treatment does not change apoptosis in intra-study BT142 treated tumors. (**e-f**) MRK-A treatment does not alter nestin levels in BT142 samples at the end of long-term study.

**Supplementary Figure S4.** (**a**) Western blot analysis using the mutant IDH1 R132H-specific antibody was conducted on GB10, BT142, MOG-R132H (positive control), and as a negative control HT1080 (which harbors a R132C mutation). (**b-d**) Western blot quantitation of IDH1 R132H protein levels relative to the beta-actin loading control.

**Supplementary Figure S5.** (**a-b**) Volcano plots of GB10 vs vehicle and BT142 vs vehicle displaying the decreased transcriptional output of GB10. Volcano plot generated in OmicSoft. Y-axis is log_10_ (*P*-value) and x-axis is log2 fold change. Note the different scale of the axis between the groups and the low range of fold changes seen in the GB10 arm. (**c**) Principal component analysis of sample groups: Principal component analysis (PCA) is a technique used to emphasize variation. It can be used as a QC step to see if the sample groups separate according to prior knowledge.

**Supplementary Figure S6.** Unbound levels of MRK-A in brain and blood of naïve C57BL6 mice.

**Supplementary Table S1.** Sequencing of the IDH1 mutant GB10 glioma model for genetic characterization.

**Supplementary Table S2.** (**a**) GB10 MRK-A vs vehicle gene expression differences

(**b**) BT142 MRK-A vs vehicle gene expression differences

**Supplementary Figure S1**

**
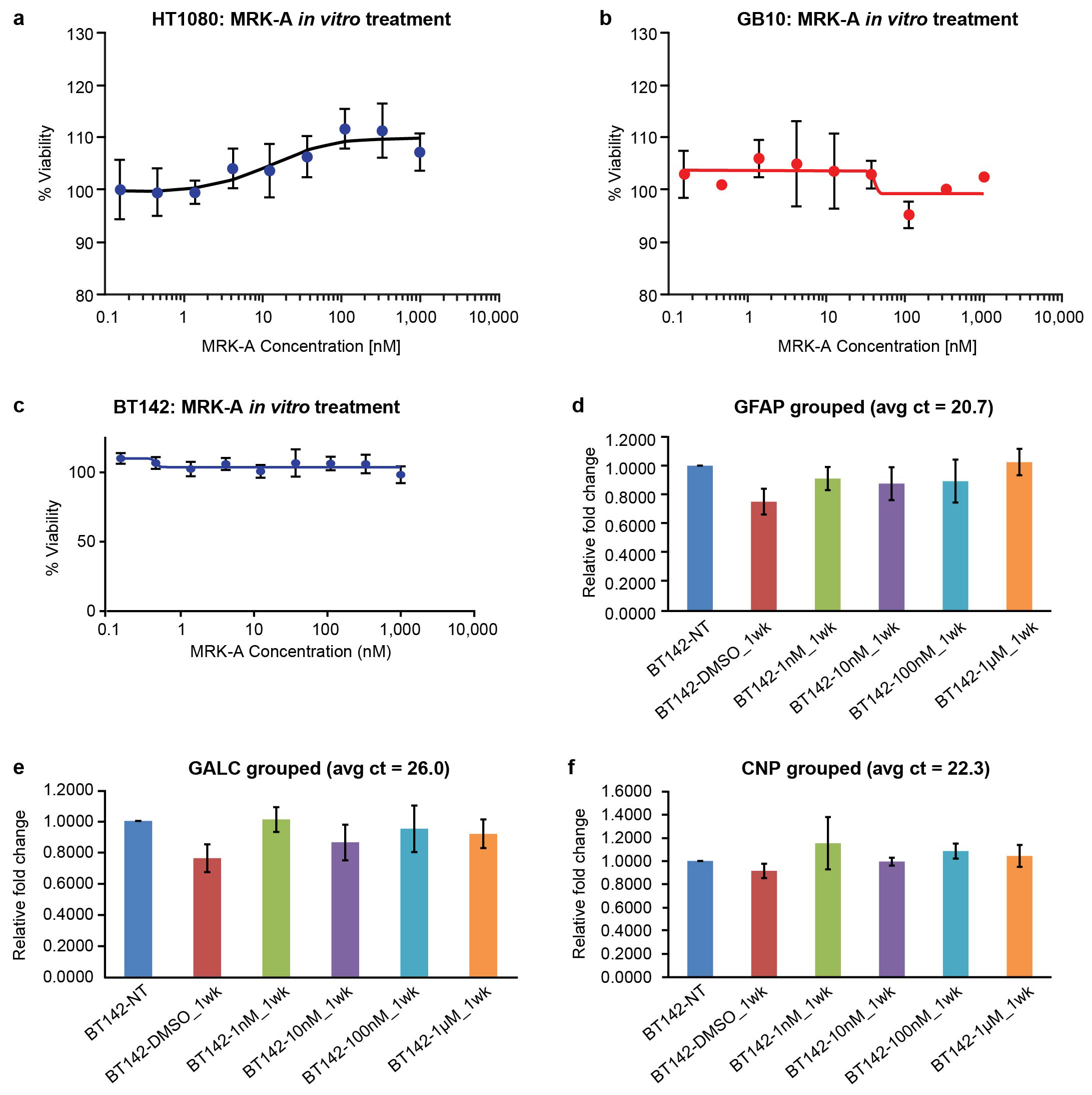
**

**Supplementary Figure S2**

**
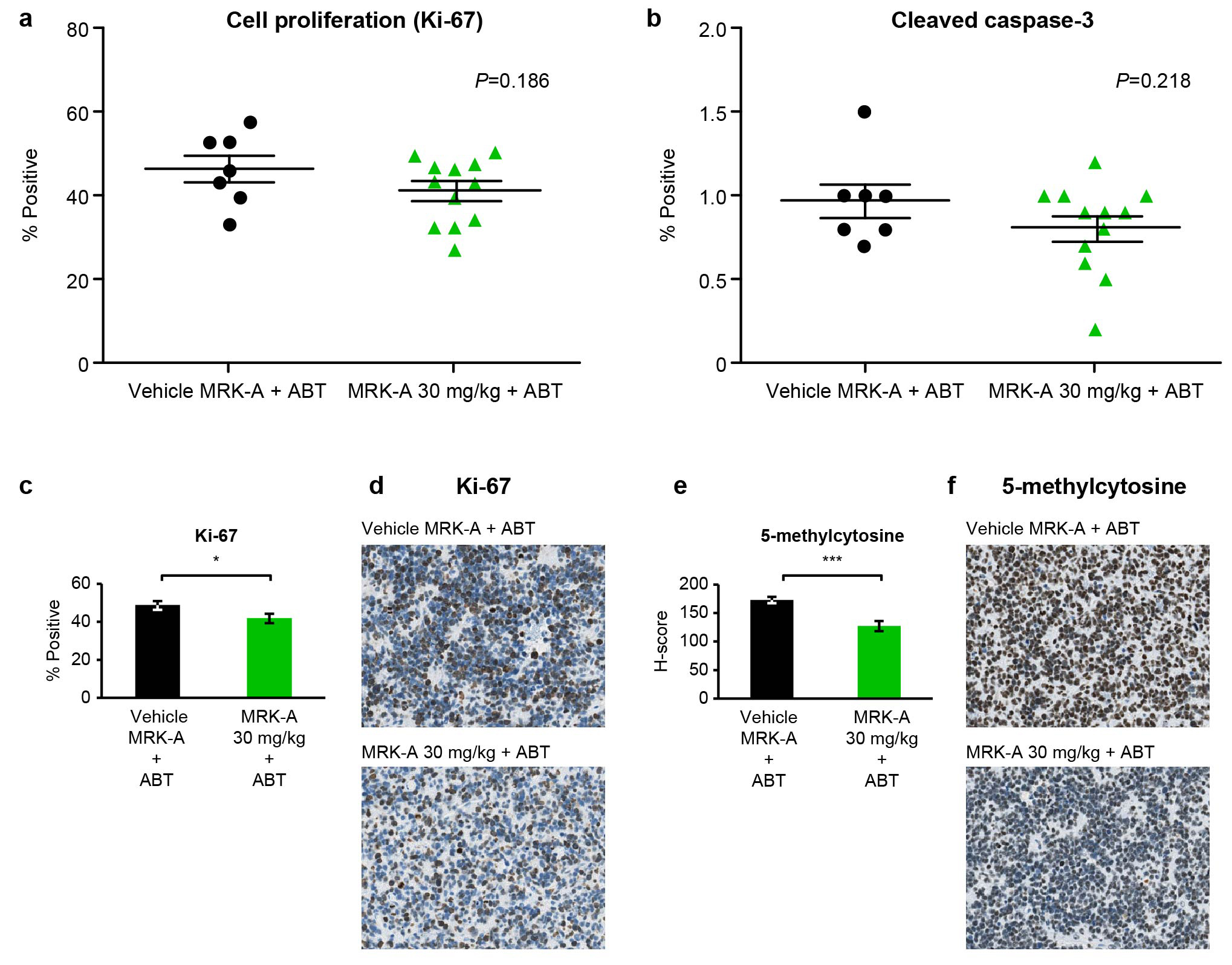
**

**Supplementary Figure S3
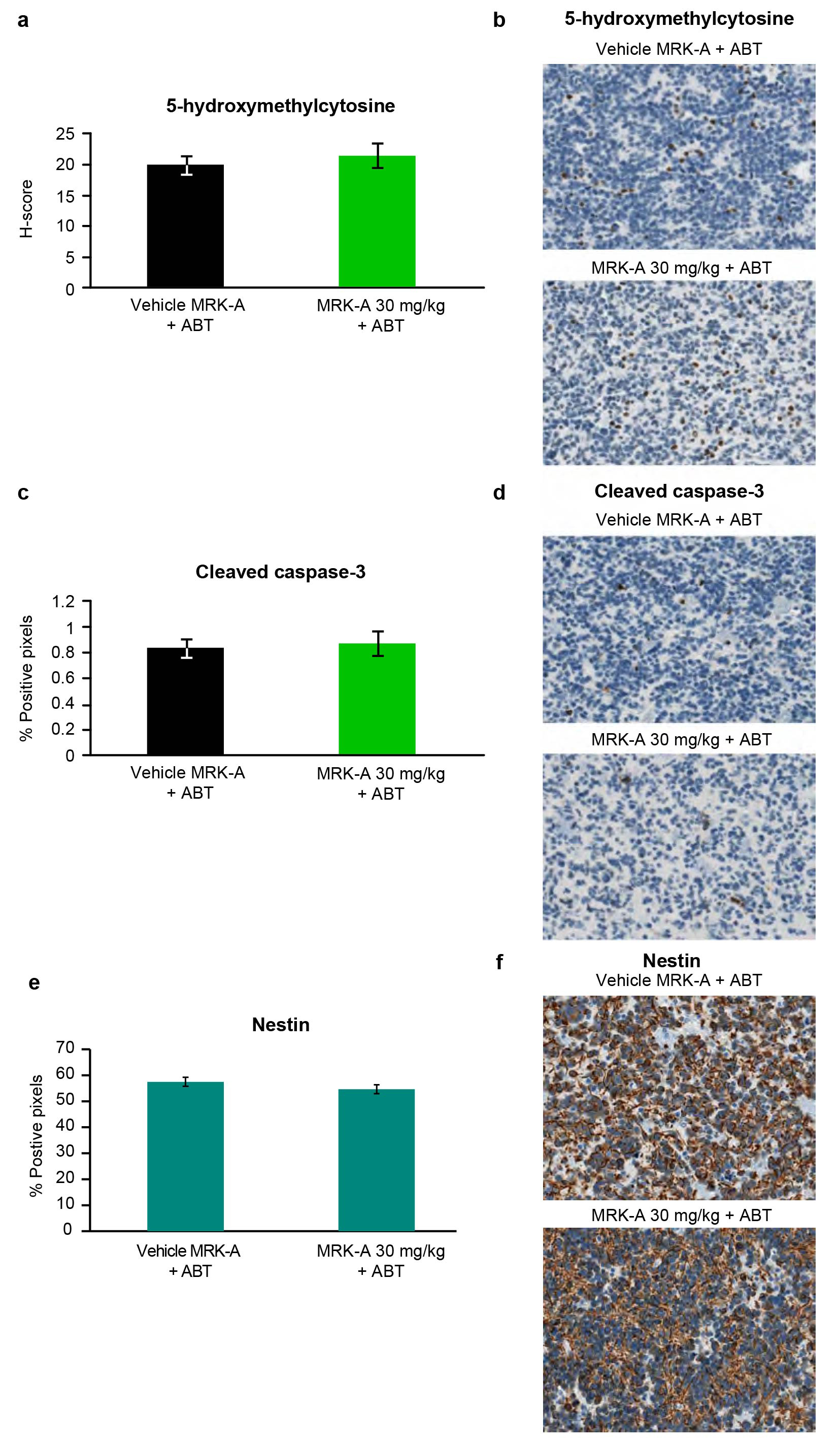
**

**Supplementary Figure S4**

**
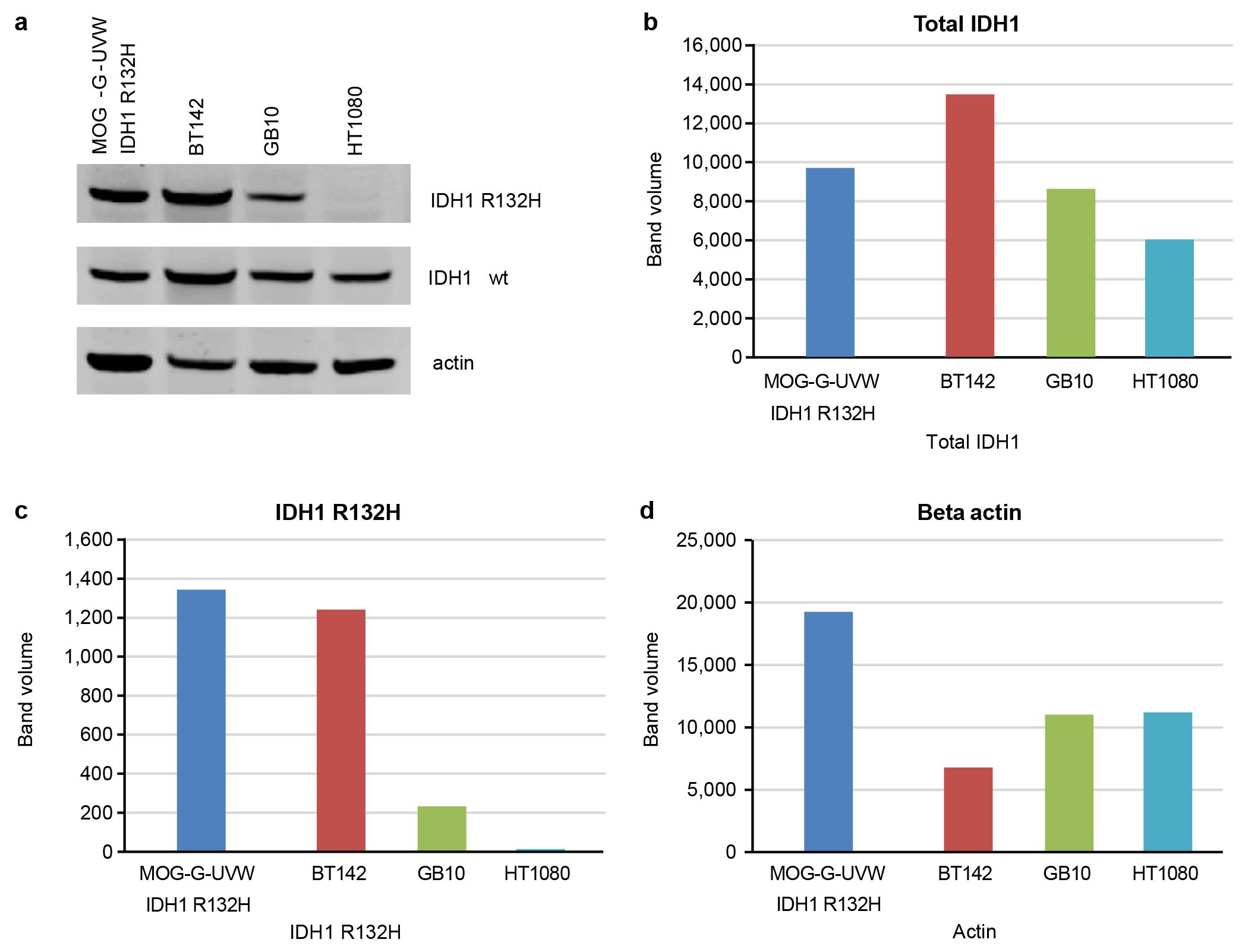
**

**Supplementary Figure S5**

**
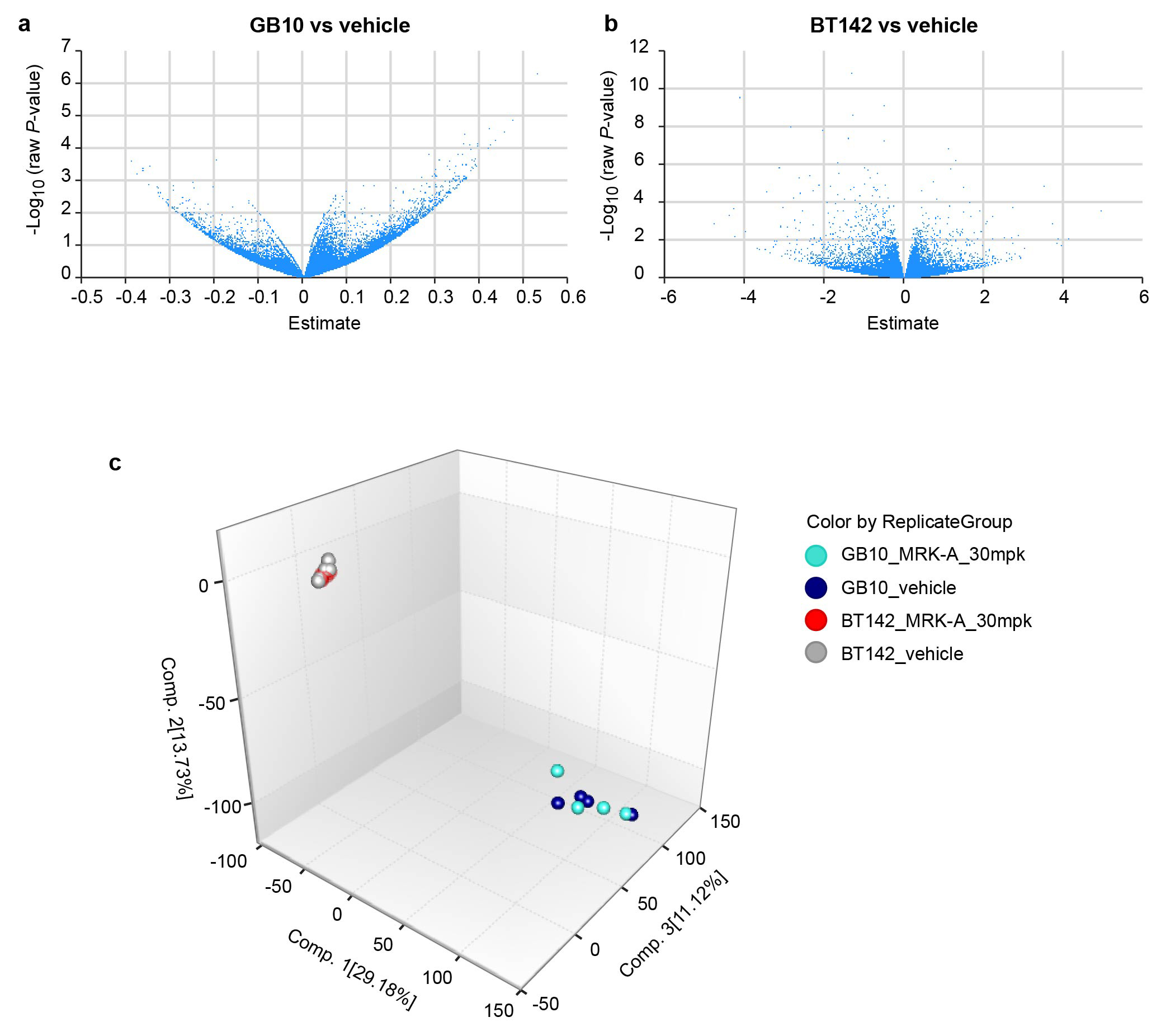
**

**Supplementary Figure S6**

**
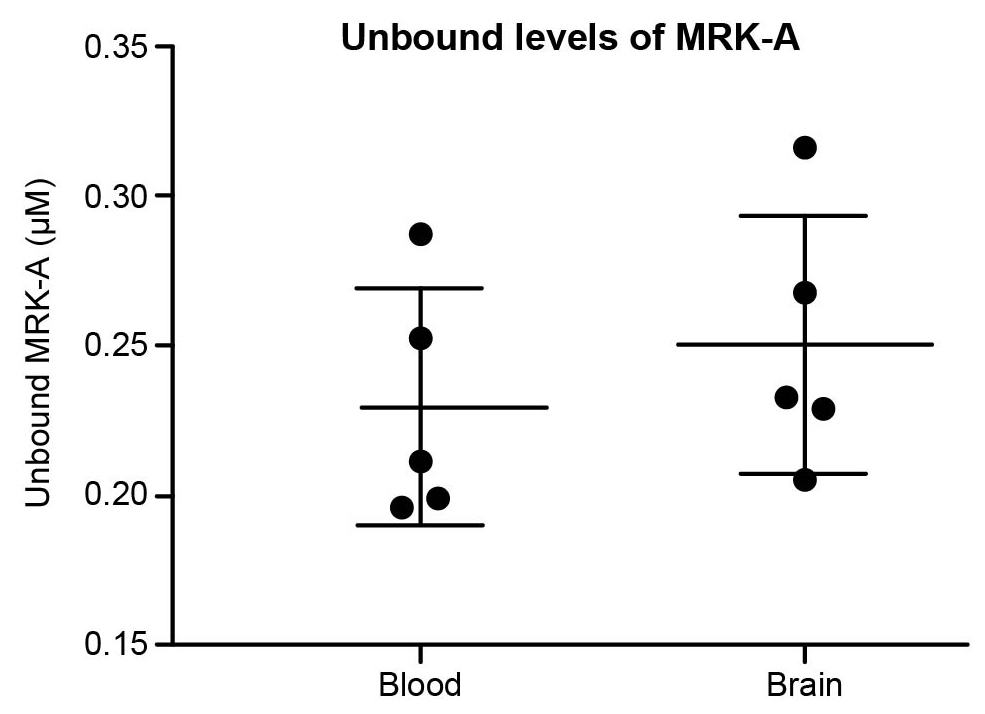
**

**Supplementary Table S1.** Sequencing of the IDH1 mutant GB10 glioma model for genetic characterization

| **Sample ID** | **Gene Name** | **Exon** | **Alleles** | **Flanking Sequence** | **Allele 1** | **Allele 2** | **Peptide Flanking Sequence** | **Mutation Type** |
| --- | --- | --- | --- | --- | --- | --- | --- | --- |
| GB-10 | IDH1 | 4 | GA | CTATCATCATAGGTCGTCATGCTTATGGGGA | R | H | PIIIGRHAYGD | *c.*g395a *p.*R132H |
| GB-10 | TP53 | 7 | TT | AACAGCTTTGAGGTGCGTGTTTGTGCCTGTC | C | C | NSFEVRVCACP | *c.*c817T *p.*R273C |
| GB-10 | PTEN |  | WT |  | WT | WT |  | N/A |
| GB-10 | FUBP1 |  | WT |  | WT | WT |  | N/A |
| GB-10 | CIC |  | WT |  | WT | WT |  | N/A |
| GB-10 | PIK3CA | 9, 20 | WT |  | WT | WT |  | N/A |

**Supplementary Table 2**

(**a**) GB10 MRK-A vs vehicle gene expression differences

| **Sample ID** | **Fold Change** |  |
| --- | --- | --- |
|  | **GB10_vehicle vs GB10_MRKA_30mpk** | ***P*-value** |
| HES4 | 1.3715 | 3.39E-05 |
| AGRN | 1.3097 | 0.0002 |
| ACAP3 | 1.2683 | 0.0008 |
| DVL1 | 1.2254 | 0.0056 |
| MIB2 | 1.2766 | 0.0013 |
| PRKCZ | 1.2587 | 0.0018 |
| AJAP1 | 1.1526 | 0.0068 |
| NPHP4 | 1.297 | 7.84E-05 |
| CHD5 | 1.2506 | 0.0034 |
| AK125437 | 1.2555 | 0.0022 |
| ALDH4A1 | 1.2338 | 0.0038 |
| SYTL1 | 1.3034 | 0.0002 |
| MAP3K6 | 1.2199 | 0.0047 |
| SNHG3 | -1.2377 | 0.0052 |
| BAI2 | 1.2366 | 0.0027 |
| MED8 | -1.2714 | 0.0004 |
| ITGB3BP | 1.2163 | 0.0092 |
| HS2ST1 | 1.1919 | 0.0083 |
| SCARNA2 | 1.3129 | 0.0004 |
| GSTM2 | -1.2988 | 0.0006 |
| DKFZp547A023 | -1.1873 | 0.0097 |
| DCLRE1B | -1.2139 | 0.0054 |
| TXNIP | -1.2236 | 0.0079 |
| GPR89B | 1.2991 | 0.0005 |
| HIST2H3D | 1.2532 | 0.0031 |
| HIST2H2AC | 1.2337 | 0.0016 |
| LELP1 | 1.0444 | 0.0061 |
| JTB | 1.2302 | 0.0005 |
| RPS27 | 1.1461 | 0.0058 |
| TDRD10 | 1.0382 | 0.0097 |
| ADAM15 | 1.2501 | 0.0024 |
| RFWD2 | 1.2296 | 0.0007 |
| AK096718 | 1.1161 | 0.0014 |
| SDE2 | -1.2037 | 0.0099 |
| **Sample ID** | **Fold Change** | ***P*-value** |
|  | **GB10_vehicle vs GB10_MRKA_30mpk** |  |
| ACBD3 | -1.2026 | 0.0042 |
| EXOC8 | -1.2088 | 0.0051 |
| AK123393 | 1.098 | 0.0014 |
| RSU1 | -1.2271 | 0.0071 |
| UNC5B | 1.2057 | 0.0071 |
| CHST3 | 1.3389 | 2.52E-05 |
| HTRA1 | 1.2005 | 0.0076 |
| METTL10 | 1.276 | 0.0014 |
| B4GALNT4 | 1.2827 | 0.0003 |
| DEAF1 | 1.224 | 0.0041 |
| PIDD | 1.2091 | 0.0073 |
| MUC2 | 1.2034 | 0.0054 |
| BRSK2 | 1.2692 | 0.001 |
| CDKN1C | 1.2906 | 0.0008 |
| PDE3B | 1.2284 | 0.0033 |
| CNTF | -1.1458 | 0.0002 |
| FAM111A | -1.2851 | 0.0004 |
| TMEM132A | 1.2379 | 0.0024 |
| SYT7 | 1.2845 | 0.001 |
| EML3 | 1.2281 | 0.0049 |
| RTN3 | 1.1532 | 0.0044 |
| C11orf95 | 1.2661 | 0.0006 |
| PPP1R14B | 1.1858 | 0.0087 |
| ESRRA | 1.2893 | 0.0003 |
| NRXN2 | 1.2145 | 0.0078 |
| LTBP3 | 1.2291 | 0.0038 |
| FAM89B | 1.2236 | 0.0028 |
| CCDC85B | 1.2602 | 0.0023 |
| CTSF | 1.23 | 0.0058 |
| TCIRG1 | 1.2357 | 0.0056 |
| CHKA | 1.2182 | 0.0041 |
| INPPL1 | 1.2433 | 0.0035 |
| ATM | 1.2423 | 0.0015 |
| MFRP | 1.2664 | 0.0019 |
| TP53AIP1 | -1.1948 | 0.0096 |
| B3GAT1 | 1.2169 | 0.0093 |
| TSPAN9 | 1.2386 | 0.0047 |
| SCARNA12 | 1.2874 | 0.0007 |
| SLC38A2 | 1.1814 | 0.0041 |
| **Sample ID** | **Fold Change** | ***P*-value** |
|  | **GB10_vehicle vs GB10_MRKA_30mpk** |  |
| KCNH3 | 1.2569 | 0.0024 |
| RARG | 1.2747 | 0.0014 |
| RAP1B | 1.1947 | 0.0077 |
| BC045559 | 1.0564 | 0.0063 |
| CHPT1 | 1.2585 | 0.0011 |
| KCTD10 | -1.2261 | 0.0014 |
| AX747754 | -1.2283 | 0.0063 |
| AK096932 | -1.2198 | 0.0069 |
| CCDC64 | 1.2369 | 0.0045 |
| FZD10 | 1.0607 | 0.0095 |
| PXMP2 | 1.2218 | 0.0045 |
| LINC00442 | 1.0523 | 0.0089 |
| DLEU2 | 1.2009 | 0.009 |
| MRPS31P5 | -1.2374 | 0.004 |
| ANKRD10 | 1.2997 | 0.0001 |
| TFDP1 | 1.2011 | 0.0021 |
| RPPH1 | 1.2648 | 0.0017 |
| AX747992 | 1.0483 | 0.0046 |
| ZNF219 | 1.274 | 0.0007 |
| BCL2L2-PABPN1 | 1.2247 | 0.0008 |
| PABPN1 | 1.1586 | 0.0087 |
| SLC22A17 | 1.2028 | 0.0065 |
| JPH4 | 1.2329 | 0.0007 |
| RPS29 | 1.2652 | 0.0009 |
| KCNK10 | 1.2174 | 0.0084 |
| SPATA7 | -1.2201 | 0.0089 |
| NRDE2 | -1.2752 | 0.0014 |
| CCDC85C | 1.2676 | 0.001 |
| EVL | 1.2029 | 0.0036 |
| YY1 | 1.2049 | 0.0008 |
| SLC25A29 | 1.2786 | 0.001 |
| ANKRD9 | 1.3877 | 1.46E-05 |
| JAG2 | 1.2826 | 0.0002 |
| BRF1 | 1.1951 | 0.0068 |
| MTA1 | 1.22 | 0.0016 |
| GABRA5 | 1.075 | 0.0052 |
| DPH6 | -1.2502 | 0.0023 |
| BAHD1 | 1.2848 | 7.23E-05 |
| DUT | 1.2274 | 0.0006 |
| **Sample ID** | **Fold Change** | ***P*-value** |
|  | **GB10_vehicle vs GB10_MRKA_30mpk** |  |
| BC034927 | 1.0509 | 0.0031 |
| LMAN1L | 1.2574 | 0.0023 |
| CPLX3 | 1.2253 | 0.0074 |
| MESDC1 | 1.2174 | 0.0065 |
| LOC388152 | 1.2338 | 0.0053 |
| MESP1 | 1.2328 | 0.0032 |
| HBA2 | 1.0521 | 0.004 |
| RAB11FIP3 | 1.2281 | 0.006 |
| PKD1 | 1.2314 | 0.0016 |
| FLJ00285 | 1.1814 | 0.0039 |
| AK304826 | 1.1997 | 0.0094 |
| ERI2 | -1.2243 | 0.0078 |
| GPT2 | 1.2044 | 0.0015 |
| CRNDE | 1.2381 | 0.0041 |
| DOK4 | 1.2025 | 0.0099 |
| FBXL8 | 1.21 | 0.0063 |
| AK055364 | -1.2473 | 0.0035 |
| CDH15 | 1.1875 | 0.0086 |
| SPIRE2 | 1.3035 | 0.0004 |
| BC139719 | 1.0683 | 0.0022 |
| SGSM2 | 1.2035 | 0.0039 |
| ZFP3 | -1.31 | 0.0003 |
| ZNF287 | -1.2398 | 0.0048 |
| SLC47A1 | 1.1966 | 0.0094 |
| DHRS13 | 1.2231 | 0.0076 |
| SUZ12P1 | 1.2122 | 0.0012 |
| RHBDL3 | 1.2693 | 0.0018 |
| SRCIN1 | 1.2541 | 0.0004 |
| MLLT6 | 1.185 | 0.0069 |
| IGFBP4 | 1.2747 | 0.0002 |
| PLEKHH3 | 1.2107 | 0.0093 |
| RPL27 | 1.1718 | 0.0082 |
| HDAC5 | 1.2249 | 0.004 |
| HEXIM1 | -1.2532 | 0.003 |
| AKAP1 | 1.1888 | 0.0018 |
| CACNG4 | 1.2339 | 0.0051 |
| TBC1D16 | 1.2094 | 0.0066 |
| PYCR1 | 1.18 | 0.0088 |
| RAB40B | 1.2171 | 0.0099 |
| **Sample ID** | **Fold Change** | ***P*-value** |
|  | **GB10_vehicle vs GB10_MRKA_30mpk** |  |
| SALL3 | -1.0825 | 0.0058 |
| CDC34 | 1.2223 | 0.0044 |
| PALM | 1.2367 | 0.0027 |
| MUM1 | 1.2313 | 0.0013 |
| APC2 | 1.2549 | 0.0025 |
| MEX3D | 1.3095 | 0.0003 |
| TCF3 | 1.2093 | 0.004 |
| CSNK1G2 | 1.2034 | 0.0082 |
| THOP1 | 1.2768 | 0.0008 |
| AES | 1.1788 | 0.0095 |
| GNA11 | 1.2161 | 0.0077 |
| MAP2K2 | 1.2172 | 0.0033 |
| UBXN6 | 1.2067 | 0.0045 |
| HDGFRP2 | 1.1975 | 0.0052 |
| PTPRS | 1.2083 | 0.0043 |
| NRTN | 1.2449 | 0.0038 |
| SAMD1 | 1.2305 | 0.0039 |
| CRTC1 | 1.2196 | 0.0081 |
| LRP3 | 1.238 | 0.0037 |
| USF2 | 1.2393 | 0.0015 |
| POU2F2 | 1.2281 | 0.0061 |
| PPP1R37 | 1.2178 | 0.0062 |
| DACT3 | 1.336 | 0.0001 |
| TMEM160 | 1.2376 | 0.0028 |
| GLTSCR1 | 1.2448 | 0.004 |
| PTOV1 | 1.2011 | 0.007 |
| RPS9 | 1.1717 | 0.0097 |
| RFPL4A | 1.0643 | 0.0093 |
| TRAPPC12 | 1.2168 | 0.0034 |
| LOC727982 | 1.0656 | 0.0038 |
| ASAP2 | 1.2034 | 0.0049 |
| SDC1 | 1.2837 | 0.001 |
| ZNF512 | -1.1762 | 0.0076 |
| PRORSD1P | -1.2261 | 0.0021 |
| CNRIP1 | 1.2173 | 0.0016 |
| EMX1 | 1.2246 | 0.0073 |
| RPL31 | 1.2087 | 0.01 |
| CCDC74B | 1.2444 | 0.0041 |
| SP9 | 1.2546 | 0.0025 |
| **Sample ID** | **Fold Change** | ***P*-value** |
|  | **GB10_vehicle vs GB10_MRKA_30mpk** |  |
| RFTN2 | -1.0789 | 0.0071 |
| OBSL1 | 1.226 | 0.0065 |
| ALPPL2 | 1.0511 | 0.0069 |
| EFHD1 | 1.2184 | 0.0094 |
| SCLY | 1.2236 | 0.0082 |
| HES6 | 1.2186 | 0.0092 |
| TRMT6 | -1.255 | 0.0017 |
| NECAB3 | 1.2587 | 0.0015 |
| TTPAL | -1.2074 | 0.0033 |
| STK4 | -1.1867 | 0.007 |
| ELMO2 | -1.1985 | 0.0044 |
| CEBPB | 1.2895 | 0.0009 |
| RPS21 | 1.2187 | 0.0041 |
| COL20A1 | -1.0745 | 0.0088 |
| ZNF512B | 1.1763 | 0.0088 |
| DIP2A | 1.1801 | 0.0084 |
| ARVCF | 1.2466 | 0.0031 |
| ZNF280B | -1.2278 | 0.0071 |
| CACNA1I | 1.2707 | 0.0015 |
| GRAMD4 | 1.2691 | 0.0004 |
| TRABD | 1.2332 | 0.006 |
| MAPK11 | 1.2128 | 0.0093 |
| ZNF621 | -1.2018 | 0.0094 |
| VIPR1 | 1.2853 | 0.0008 |
| ZNF660 | -1.2871 | 0.0005 |
| PLXNB1 | 1.2314 | 0.0026 |
| BOC | 1.2076 | 0.004 |
| PLXNA1 | 1.2569 | 0.0003 |
| PODXL2 | 1.2079 | 0.0054 |
| H1FX | 1.2627 | 0.001 |
| PLXND1 | 1.1987 | 0.0088 |
| C3orf80 | 1.1866 | 0.0043 |
| TMEM207 | 1.0596 | 0.0051 |
| MFI2 | 1.2862 | 0.0009 |
| IDUA | 1.2311 | 0.005 |
| MXD4 | 1.2778 | 0.0011 |
| HMX1 | 1.4439 | 5.26E-07 |
| PCDH7 | 1.2112 | 0.0023 |
| RPL9 | 1.2382 | 0.0002 |
| **Sample ID** | **Fold Change** | ***P*-value** |
|  | **GB10_vehicle vs GB10_MRKA_30mpk** |  |
| SLC10A4 | 1.2632 | 0.0011 |
| HPGDS | 1.048 | 0.0037 |
| RPL34 | 1.2874 | 3.96E-05 |
| SEC24D | -1.2183 | 0.0094 |
| PDE5A | -1.1522 | 0.0015 |
| RPS3A | 1.1685 | 0.0025 |
| CPE | 1.1706 | 0.0082 |
| LRRC14B | 1.2391 | 0.005 |
| CTNND2 | 1.2005 | 0.0086 |
| BASP1 | 1.1953 | 0.0037 |
| ATG10 | -1.221 | 0.0087 |
| NDFIP1 | 1.1788 | 0.0044 |
| FAXDC2 | -1.1858 | 0.0017 |
| ERGIC1 | 1.2053 | 0.0065 |
| UNC5A | 1.2176 | 0.0091 |
| LOC100132062_2 | 1.0937 | 0.0074 |
| FOXC1 | 1.2429 | 0.0042 |
| FAM217A | 1.0392 | 0.009 |
| HIST1H4B | 1.2699 | 0.0011 |
| HIST1H3B | 1.2452 | 0.0016 |
| HIST1H2BB | 1.3071 | 0.0003 |
| HIST1H4C | 1.3136 | 8.75E-05 |
| HIST1H2AD | 1.2451 | 0.0021 |
| HIST1H3H | 1.2139 | 0.0083 |
| HIST1H4J | 1.2887 | 0.0009 |
| HIST1H3I | 1.249 | 0.0028 |
| ZSCAN26 | -1.2345 | 0.0058 |
| HLA-DPB2 | 1.0518 | 0.0028 |
| COL11A2 | 1.2197 | 0.0092 |
| RGL2 | 1.2307 | 0.0008 |
| PHF1 | 1.2203 | 0.0036 |
| CUTA | 1.2182 | 0.0002 |
| PACSIN1 | 1.2276 | 0.0059 |
| PTK7 | 1.2107 | 0.0044 |
| TDRD6 | -1.1894 | 0.0011 |
| COX7A2 | 1.1936 | 0.0044 |
| MARCKS | 1.1664 | 0.0077 |
| FABP7 | -1.2005 | 0.0069 |
| EYA4 | -1.117 | 0.0063 |
| **Sample ID** | **Fold Change** | ***P*-value** |
|  | **GB10_vehicle vs GB10_MRKA_30mpk** |  |
| CNKSR3 | -1.2589 | 0.0016 |
| NOX3 | 1.0634 | 0.0031 |
| PDE10A | -1.1606 | 0.0056 |
| DACT2 | 1.048 | 0.0047 |
| FAM20C | 1.2202 | 0.0068 |
| TNRC18 | 1.2477 | 0.0011 |
| TWISTNB | -1.2343 | 0.0056 |
| ZNRF2 | 1.2662 | 0.0006 |
| BC073780 | 1.2075 | 0.0037 |
| CLIP2 | 1.2573 | 0.001 |
| ZKSCAN5 | -1.1849 | 0.0055 |
| PRRT4 | 1.2117 | 0.0073 |
| LUC7L2 | 1.1886 | 0.0053 |
| LOC154761 | 1.0633 | 0.0091 |
| DEFA1 | 1.0416 | 0.0083 |
| XKR6 | 1.2222 | 0.0079 |
| FAM66D | 1.1004 | 0.0072 |
| TUSC3 | 1.1898 | 0.0064 |
| HR | 1.302 | 0.0004 |
| BMP1 | 1.2398 | 0.004 |
| RPS20 | 1.1573 | 0.0018 |
| DPY19L4 | 1.2014 | 0.0044 |
| MED30 | 1.1917 | 0.0069 |
| NDUFB9 | 1.1692 | 0.0023 |
| KCNK9 | 1.2573 | 0.0023 |
| AK311257 | 1.3411 | 7.93E-05 |
| LY6E | 1.2665 | 0.0006 |
| ZNF696 | 1.2061 | 0.0062 |
| SCRIB | 1.314 | 7.56E-05 |
| MAF1 | 1.2576 | 0.0005 |
| MROH1 | 1.2296 | 0.0047 |
| BOP1 | 1.2136 | 0.0078 |
| SLC52A2 | 1.2105 | 0.0088 |
| CYHR1 | 1.2234 | 0.0066 |
| KIFC2 | 1.2767 | 0.0009 |
| PPP1R16A | 1.3497 | 5.89E-05 |
| GPT | 1.2706 | 0.0017 |
| MFSD3 | 1.2385 | 0.0051 |
| RECQL4 | 1.2425 | 0.0038 |
| **Sample ID** | **Fold Change** | ***P*-value** |
|  | **GB10_vehicle vs GB10_MRKA_30mpk** |  |
| C8orf82 | 1.2037 | 0.0073 |
| ARHGAP39 | 1.2721 | 0.001 |
| CDKN2A | 1.233 | 0.0023 |
| PLAA | -1.197 | 0.0095 |
| PRPF4 | -1.236 | 0.0039 |
| OR1Q1 | 1.0518 | 0.0036 |
| SH3GLB2 | 1.1889 | 0.0098 |
| RALGDS | 1.1982 | 0.0055 |
| ABCA2 | 1.253 | 0.0024 |
| ZMYND19 | 1.2401 | 0.0023 |
| RPL9_2 | -1.0883 | 0.005 |
| CXorf38 | -1.2181 | 0.0071 |
| NDUFB11 | 1.1915 | 0.0046 |
| SNORA11D | -1.0891 | 0.0044 |
| ERCC6L | -1.2235 | 0.0067 |
| SMARCA1 | -1.2547 | 0.0024 |
| ATP11C | 1.2102 | 0.007 |
| CSAG3 | -1.1948 | 0.004 |

(**b**) BT142 MRK-A vs vehicle gene expression differences

| **Vehicle ID** | **Fold Change** | ***P*-value** |
| --- | --- | --- |
|  | **BT142 VEHICLE vs MRKA 30 mpk** |  |
| AK125437 | -1.3436 | 0.0085 |
| FLJ37453 | 1.406 | 0.0012 |
| ALPL | -3.729 | 0.0004 |
| EPHB2 | -1.6946 | 1.5E-05 |
| AK025975 | -2.1032 | 0.0002 |
| TMEM57 | -1.1806 | 0.0066 |
| UBXN11 | 1.494 | 0.0034 |
| GPR3 | -1.8962 | 0.0056 |
| LAPTM5 | -4.835 | 2.47E-05 |
| FABP3 | -2.2927 | 0.0007 |
| SERINC2 | -1.4835 | 0.0078 |
| SPATA6 | -5.8462 | 0.0003 |
| RAB3B | 1.5276 | 0.0007 |
| DNAJC6 | -1.38 | 0.0013 |
| LMO4 | -1.2979 | 0.0081 |
| FNBP1L | -1.2907 | 0.0088 |
| ARHGAP29 | 2.6503 | 0.0048 |
| TMEM56 | -3.2866 | 0.004 |
| KIAA1324 | -1.7918 | 0.0058 |
| MYBPHL | -2.3438 | 7.1E-05 |
| OLFML3 | -2.505 | 0.0072 |
| SLC22A15 | -1.8904 | 0.0081 |
| FMO5 | -1.5636 | 0.0065 |
| SELENBP1 | -2.2856 | 0.0016 |
| S100A6 | -1.772 | 0.0001 |
| S100A16 | -1.449 | 0.0004 |
| S100A13 | -2.6477 | 3.99E-08 |
| CHRNB2 | -1.5588 | 0.0019 |
| C1orf85 | -3.06 | 0.0019 |
| RRNAD1 | 1.4316 | 0.0034 |
| IFI16 | -2.4648 | 0.008 |
| CADM3 | -3.4639 | 0.0045 |
| TAGLN2 | -1.3314 | 0.0076 |
| ATP1A2 | -7.2166 | 9.62E-09 |
| **Vehicle ID** | **Fold Change** | **P-value** |
|  | **BT142 VEHICLE vs MRKA 30 mpk** |  |
| PRRX1 | -1.2512 | 0.0016 |
| KLHL20 | -1.212 | 0.0029 |
| FAM5B | 2.8172 | 1.6E-05 |
| MR1 | -1.5501 | 0.0059 |
| RGS8 | -3.1096 | 3.03E-06 |
| FAM129A | -1.4293 | 0.0015 |
| CFHR1 | -2.0831 | 0.0096 |
| CSRP1 | -1.4134 | 0.0014 |
| PPFIA4 | -1.5157 | 0.0089 |
| CHI3L1 | -8.3033 | 0.0002 |
| CNTN2 | -4.2855 | 0.0087 |
| KCNK2 | 1.2316 | 0.0046 |
| CNIH3 | -1.9456 | 0.0003 |
| DUSP5P1 | -2.434 | 0.0089 |
| ACTN2 | 1.7114 | 0.008 |
| PRTFDC1 | -1.2794 | 0.0078 |
| ANK3 | -1.4117 | 0.0006 |
| SPOCK2 | -2.7392 | 0.0012 |
| MARVELD1 | -1.679 | 0.005 |
| AFAP1L2 | -1.8283 | 0.006 |
| ZRANB1 | -1.1951 | 0.008 |
| FAM196A | -2.0957 | 0.0032 |
| NRIP3 | -2.1344 | 0.0005 |
| DCDC1 | 14.5471 | 0.0075 |
| SLC1A2 | -1.4654 | 0.0069 |
| SYT13 | -4.1274 | 1.44E-08 |
| FAM111A | 1.1599 | 0.0068 |
| RASGRP2 | -1.6687 | 0.0061 |
| NEAT1 | -1.8574 | 1.8E-06 |
| PGM2L1 | -1.4529 | 0.0006 |
| PAK1 | -1.609 | 2.76E-06 |
| CTSC | -1.7976 | 0.0008 |
| FAM76B | -1.1676 | 0.0092 |
| CASP4 | -6.2561 | 5.43E-06 |
| GRIA4 | -1.4078 | 8.33E-10 |
| TNFRSF1A | -1.8622 | 0.0055 |
| LTBR | -6.4643 | 0.0093 |
| CLSTN3 | -1.8194 | 0.0005 |
| AK096314 | -2.4272 | 2.48E-09 |
| **Vehicle ID** | **Fold Change** | ***P*-value** |
|  | **BT142 VEHICLE vs MRKA 30 mpk** |  |
| KLRC4 | -1.9232 | 0.0002 |
| KLRC3 | -1.4949 | 0.0032 |
| KLRC2 | -1.6225 | 0.0006 |
| KLRC1 | -10.889 | 2.9E-05 |
| CDKN1B | -1.2764 | 0.0009 |
| KIAA1467 | -1.6775 | 2.13E-05 |
| ATF7IP | -1.37 | 1.18E-05 |
| MGP | -3.5995 | 0.0019 |
| PLEKHA5 | -1.6882 | 0.0006 |
| AX746523 | -1.9449 | 0.0002 |
| NELL2 | -1.7605 | 0.0079 |
| TUBA1C | -1.6098 | 0.0048 |
| FAIM2 | -1.4345 | 0.0023 |
| GPD1 | -3.3174 | 0.0001 |
| POU6F1 | -1.3163 | 0.0036 |
| ITGA5 | -3.4649 | 0.0013 |
| DGKA | -1.3826 | 0.0022 |
| RNF41 | -1.2201 | 0.0073 |
| NABP2 | -1.2299 | 0.0078 |
| STAT6 | -2.8291 | 6.67E-05 |
| DDIT3 | -1.4288 | 0.0067 |
| PIP4K2C | -1.4669 | 0.0023 |
| CTDSP2 | -1.1913 | 0.0081 |
| KITLG | 1.724 | 0.0007 |
| ANKS1B | -1.3897 | 0.0011 |
| NUAK1 | -1.5297 | 0.0022 |
| RPH3A | -1.7432 | 0.0042 |
| AK096932 | -1.9754 | 1.51E-06 |
| HRK | 2.3426 | 0.0059 |
| CLIP1 | -1.2948 | 0.0002 |
| HCAR1 | -19.2665 | 0.0002 |
| SCARB1 | 1.6529 | 0.0028 |
| GALNT9 | 4.195 | 0.001 |
| AX747752 | 6.4707 | 0.0067 |
| LOC100130238 | 13.1097 | 0.0013 |
| PXMP2 | 1.2735 | 0.0077 |
| SAP18 | -1.1981 | 0.0014 |
| TPTE2P1 | -1.6269 | 0.0013 |
| SLAIN1 | -1.4533 | 0.0003 |
| **Vehicle ID** | **Fold Change** | ***P*-value** |
|  | **BT142 VEHICLE vs MRKA 30 mpk** |  |
| ZIC5 | 4.1059 | 0.0014 |
| FGF14 | -1.551 | 0.0019 |
| LINC00346 | -3.1871 | 0.0062 |
| AP1G2 | 1.4314 | 0.0092 |
| LINC00645 | -2.1899 | 0.0046 |
| AKAP6 | -1.3248 | 0.0073 |
| LRFN5 | 2.7894 | 0.0003 |
| AK055910 | -1.4183 | 0.0067 |
| LTBP2 | -6.5323 | 0.0022 |
| FAM181A | -6.0518 | 0.0011 |
| KIF26A | 2.4885 | 0.0036 |
| THBS1 | 2.4981 | 0.0026 |
| PLCB2 | -2.6262 | 0.0008 |
| CHAC1 | 1.8148 | 0.0053 |
| STARD9 | 1.1778 | 0.0046 |
| CKMT1A | -3.6514 | 0.0012 |
| SERF2-C15ORF63 | 1.373 | 0.0058 |
| SLC12A1 | -4.3359 | 0.0066 |
| SCG3 | -1.4092 | 5.36E-08 |
| MYO1E | 1.8852 | 0.003 |
| ANXA2 | -1.5933 | 1.96E-05 |
| LARP6 | -1.2842 | 0.004 |
| CRABP1 | -1.6812 | 0.0042 |
| GOLGA6L4 | -3.4486 | 0.0082 |
| FES | -2.5944 | 0.0057 |
| CACNA1H | 3.8395 | 0.0034 |
| SNORA10 | -1.913 | 0.0026 |
| LOC100507501 | 4.7106 | 0.0076 |
| AK057657 | -1.2611 | 0.0032 |
| EMP2 | -3.075 | 0.0075 |
| DEXI | -1.3301 | 0.009 |
| SNN | -1.2223 | 0.0049 |
| ARL6IP1 | -1.1517 | 0.0076 |
| NUPR1 | -3.1938 | 8.07E-07 |
| SULT1A3 | -2.3974 | 0.0029 |
| KCTD13 | -1.4762 | 0.0059 |
| FUS | 1.2228 | 0.0099 |
| WWP2 | -1.3826 | 0.0004 |
| AX747598 | -2.0977 | 0.0018 |
| **Vehicle ID** | **Fold Change** | **P-value** |
|  | **BT142 VEHICLE vs MRKA 30 mpk** |  |
| SERPINF1 | -2.2247 | 0.0011 |
| RAP1GAP2 | -1.9681 | 0.0016 |
| WSCD1 | -1.3155 | 0.0026 |
| GAS7 | -1.8155 | 5.04E-06 |
| PLD6 | 1.4341 | 0.0033 |
| DRG2 | -3.0592 | 0.0029 |
| ULK2 | -1.4075 | 0.0004 |
| UBBP4 | -1.724 | 0.0022 |
| EVI2A | 2.2562 | 0.0053 |
| GPR179 | -2.0584 | 0.0008 |
| LINC00672 | -3.1995 | 3.75E-05 |
| ERBB2 | 1.3193 | 0.0029 |
| FAM187A | 1.607 | 0.0061 |
| NSFP1 | -4.9052 | 0.0016 |
| EFCAB13 | -1.9512 | 0.0062 |
| HOXB3 | 30.7292 | 0.0003 |
| MMD | -1.213 | 0.0045 |
| LRRC37A3_2 | -1.2848 | 0.0032 |
| ABCA9 | -3.8532 | 9.6E-05 |
| SOCS3 | -1.4734 | 0.0086 |
| WDR45B | -1.2438 | 0.0028 |
| METRNL | -2.3072 | 0.006 |
| EPB41L3 | 3.1021 | 0.0001 |
| RAB31 | -1.2281 | 0.0087 |
| DSG2 | -3.6724 | 0.0011 |
| KLHL14 | -2.8995 | 0.0035 |
| LOC100287225 | -4.2484 | 0.009 |
| ZNF532 | -1.2431 | 0.0041 |
| CDH19 | -1.7832 | 0.0002 |
| CNN2 | 1.6683 | 8.08E-06 |
| MRI1 | 2.1141 | 0.0012 |
| LOC284454 | -1.9029 | 0.0007 |
| AK022793 | -2.5602 | 0.0036 |
| AK092080 | 4.6956 | 0.0006 |
| FBXO27 | -7.6395 | 0.0057 |
| LRFN1 | 3.0484 | 0.0096 |
| TTC9B | -2.0347 | 0.002 |
| SLC8A2 | 7.359 | 0.0051 |
| ATF5 | -2.0881 | 0.0063 |
| **Vehicle ID** | **Fold Change** | ***P*-value** |
|  | **BT142 VEHICLE vs MRKA 30 mpk** |  |
| ZNF816-ZNF321P | 17.4095 | 0.0082 |
| ZNF551 | 1.2496 | 0.0072 |
| LOC400940 | -1.6185 | 0.0001 |
| FAM49A | -1.4427 | 0.0009 |
| DNMT3A | -1.2646 | 0.0054 |
| EMILIN1 | -1.9277 | 0.0002 |
| RBKS | -4.7595 | 0.0085 |
| LBH | -1.4623 | 0.0058 |
| VIT | -6.7201 | 0.0008 |
| CDC42EP3 | -1.6495 | 0.0005 |
| NRXN1 | -1.6989 | 0.0008 |
| CYP26B1 | 2.7223 | 0.0035 |
| RAB11FIP5 | -1.8961 | 0.0021 |
| TACR1 | 2.7482 | 0.0042 |
| CAPG | -3.5838 | 0.0049 |
| ARID5A | -1.4221 | 0.0053 |
| PTPN18 | -1.5594 | 0.0015 |
| TNFAIP6 | -2.3665 | 0.0006 |
| GRB14 | -1.5216 | 0.003 |
| SCN2A | -1.5959 | 0.0002 |
| SCN9A | -2.0052 | 0.0031 |
| HOXD11 | -1.6157 | 0.0015 |
| UBE2E3 | -1.1701 | 0.0076 |
| COL5A2 | -2.4095 | 1.56E-05 |
| CFLAR | -1.4064 | 0.0006 |
| CASP8 | -2.1476 | 0.0001 |
| C2orf80 | -1.5906 | 0.0092 |
| CPS1 | -2.1784 | 4.57E-05 |
| ABCA12 | -2.0495 | 0.0077 |
| SCG2 | -2.7504 | 0.0076 |
| TRPM8 | -2.4709 | 0.002 |
| CHGB | -2.3073 | 0.0003 |
| KIF3B | -1.1731 | 0.0025 |
| MMP24 | -2.2104 | 0.0005 |
| PPP1R16B | 3.7721 | 0.0003 |
| IFT52 | -1.3068 | 0.0001 |
| ZFAS1 | -1.2085 | 0.0002 |
| VAPB | 1.2023 | 0.0082 |
| SLMO2-ATP5E | 3.6019 | 0.0076 |
| **Vehicle ID** | **Fold Change** | ***P*-value** |
|  | **BT142 VEHICLE vs MRKA 30 mpk** |  |
| CHRNA4 | 2.1558 | 0.0006 |
| MYT1 | -1.3917 | 0.0058 |
| LINC00478 | -1.3543 | 0.0068 |
| LINC00189 | -27.0997 | 0.0013 |
| SLC37A1 | -5.0605 | 0.0077 |
| PEX26 | 1.2982 | 0.0032 |
| DGCR2 | 1.3545 | 0.009 |
| PI4KAP1 | 1.4148 | 0.0086 |
| RIMBP3 | 2.463 | 0.0094 |
| PI4KAP2 | 1.6774 | 0.0098 |
| TOP3B | 1.3387 | 0.0044 |
| FAM211B | 1.701 | 0.0046 |
| ZNRF3 | 1.3312 | 0.0091 |
| PATZ1 | 1.216 | 0.0095 |
| FBXO7 | 1.2516 | 0.0037 |
| HMGXB4 | 1.2667 | 0.0077 |
| MCM5 | 1.1954 | 0.0088 |
| ELFN2 | 3.1576 | 0.0006 |
| CDC42EP1 | 2.1899 | 0.0048 |
| TOMM22 | 1.2405 | 0.0032 |
| NHP2L1 | 1.257 | 0.0023 |
| RRP7A | 1.3834 | 0.0004 |
| CDPF1 | 1.6111 | 0.009 |
| MLC1 | 1.8664 | 0.0007 |
| CHKB-CPT1B | 2.5497 | 0.0039 |
| LHFPL4 | -1.3473 | 0.0065 |
| ZNF385D | 2.9774 | 0.0082 |
| UBE2E1 | -1.2071 | 0.0037 |
| RARB | -1.7013 | 0.0084 |
| IL17RB | -1.6703 | 0.0002 |
| ADAMTS9 | -1.7265 | 0.0004 |
| FRMD4B | 2.2611 | 1.6E-06 |
| CRYBG3 | 1.4905 | 0.0075 |
| SIDT1 | -20.9512 | 0.0004 |
| STXBP5L | -1.4999 | 0.0028 |
| ISY1-RAB43 | -1.5463 | 0.0022 |
| ACPL2 | -1.2927 | 0.0073 |
| ZBTB38 | -1.3596 | 0.0003 |
| GK5 | -1.2934 | 0.0082 |
| **Vehicle ID** | **Fold Change** | ***P*-value** |
|  | **BT142 VEHICLE vs MRKA 30 mpk** |  |
| TM4SF1 | -2.0251 | 0.0027 |
| PFN2 | 1.2089 | 0.0071 |
| SAMD7 | -18.889 | 0.0061 |
| MCF2L2 | -1.3721 | 0.0017 |
| B3GNT5 | -8.5418 | 0.0017 |
| ECE2 | -1.6622 | 0.0052 |
| CLCN2 | -1.7336 | 4.32E-05 |
| FGFR3 | 2.1168 | 0.01 |
| NSG1 | -1.8372 | 0.0012 |
| EVC2 | -3.9032 | 0.0052 |
| KCNIP4-IT1 | 2.2695 | 0.0091 |
| STIM2 | 1.2829 | 0.0033 |
| CHRNA9 | -2.8723 | 0.0003 |
| LIMCH1 | 1.4591 | 0.0022 |
| LNX1 | -5.1867 | 3.69E-06 |
| KDR | 6.5316 | 0.0002 |
| PPAT | 1.2278 | 0.0084 |
| PAICS | 1.2228 | 0.0076 |
| FW340024 | 1.5028 | 0.0017 |
| UNC5C | -2.0393 | 0.0025 |
| ZNF827 | 1.3274 | 0.0041 |
| CPE | -1.3188 | 5.34E-05 |
| FAT1 | 1.3657 | 0.0063 |
| CMBL | 1.4663 | 0.0004 |
| NPR3 | -2.5391 | 2.13E-05 |
| PLK2 | -1.9115 | 0.0008 |
| NDUFAF2 | 1.3662 | 0.0031 |
| GUSBP9 | 1.2957 | 0.0096 |
| MRPS27 | 1.1852 | 0.0027 |
| HOMER1 | 1.3447 | 2.47E-05 |
| ACSL6 | 2.4258 | 6.43E-07 |
| PHF15 | -1.7417 | 0.0089 |
| SPOCK1 | 1.716 | 0.0006 |
| PCDHA1 | 3.3868 | 0.0042 |
| PCDHGB1 | 1.7645 | 6.24E-05 |
| PCDHGA4 | 2.1441 | 1.5E-07 |
| PCDHGB2 | 1.7548 | 0.0056 |
| PCDHGA5 | 1.7844 | 0.0005 |
| PCDHGA8 | 1.4776 | 0.0055 |
| **Vehicle ID** | **Fold Change** | ***P*-value** |
|  | **BT142 VEHICLE vs MRKA 30 mpk** |  |
| ARAP3 | -1.7712 | 0.0032 |
| RREB1 | 2.0852 | 0.0079 |
| HIST1H2AG | 1.3229 | 0.0071 |
| HIST1H1B | 1.2279 | 0.0024 |
| HIST1H3I | 1.3523 | 0.0002 |
| CLIC1 | -1.3649 | 0.0083 |
| EYS | -3.1997 | 0.0003 |
| GRIK2 | -1.6043 | 0.0017 |
| BVES | -1.274 | 0.0067 |
| FABP7 | -1.2118 | 0.0074 |
| EPB41L2 | 1.3434 | 0.0065 |
| UTRN | 1.7131 | 0.0013 |
| PLEKHG1 | 2.2196 | 0.0002 |
| IPCEF1 | -2.3458 | 0.0017 |
| AK090788 | -15.7006 | 0.0034 |
| GLCCI1 | 1.3722 | 0.0012 |
| AHR | -1.4331 | 0.0011 |
| GPNMB | -4.4269 | 1.22E-05 |
| MPP6 | 1.8685 | 0.0071 |
| ZNF679 | -7.5639 | 0.0019 |
| DQ571357 | -8.8102 | 1.5E-06 |
| ZP3 | -8.5584 | 0.0021 |
| FGL2 | -4.8105 | 0.0069 |
| GNAI1 | 1.3331 | 0.0047 |
| SEMA3C | 1.862 | 0.0033 |
| HGF | -2.157 | 0.0057 |
| PEG10 | 1.2818 | 0.0048 |
| LOC100289561 | -1.6714 | 0.0014 |
| DPY19L2P2 | 1.3401 | 0.0058 |
| ATXN7L1 | 1.5239 | 0.0014 |
| CPED1 | 1.8805 | 0.0041 |
| DLC1 | -2.4932 | 1.39E-11 |
| LPL | -3.372 | 0.0007 |
| TEX15 | 2.7298 | 0.0026 |
| NECAB1 | -1.7867 | 0.0007 |
| ZNF706 | 1.271 | 0.0054 |
| BAALC | -1.2997 | 0.0081 |
| FZD6 | -2.6858 | 0.0016 |
| RIMS2 | -1.4554 | 0.0002 |
| **Vehicle ID** | **Fold Change** | ***P*-value** |
|  | **BT142 VEHICLE vs MRKA 30 mpk** |  |
| SYBU | -1.6569 | 0.0002 |
| NDRG1 | -1.586 | 0.0033 |
| ARC | 1.4027 | 0.002 |
| AK021739 | 2.3532 | 0.0037 |
| Metazoa_SRP_43 | -3.5798 | 1.42E-05 |
| TEK | -17.5055 | 3.07E-10 |
| MOB3B | 1.6295 | 0.0022 |
| KIF24 | 1.3132 | 0.0059 |
| RUSC2 | -1.2117 | 0.008 |
| LINC00950 | 1.4646 | 0.009 |
| GRHPR | 1.2212 | 0.009 |
| ANKRD18A | 11.4283 | 1.36E-05 |
| FAM201A | 5.7309 | 0.007 |
| RMI1 | 1.3953 | 0.0034 |
| DAPK1 | 2.6346 | 0.0056 |
| ZNF883 | -1.2292 | 0.0039 |
| MVB12B | -1.3912 | 0.001 |
| PKN3 | 1.626 | 0.0072 |
| GLRA2 | -2.5394 | 0.0019 |
| MAP7D2 | -2.6405 | 0.0066 |
| TMEM47 | 1.2989 | 0.0031 |
| MAOA | -1.8559 | 0.0011 |
| RBM3 | -1.2601 | 0.0087 |
| MAGED4B | -1.44 | 0.0023 |
| TSPYL2 | -1.3214 | 0.001 |
| DLG3 | -1.3866 | 0.0016 |
| SLC7A3 | -6.1883 | 0.003 |
| DRP2 | -1.6417 | 2.56E-05 |
| GPRASP2 | -2.1627 | 0.0014 |
| BEX1 | -1.5672 | 0.0003 |
| BEX4 | -2.0927 | 0.0002 |
| TCEAL5 | -1.7203 | 0.0043 |
| BEX2 | -2.003 | 9.82E-06 |
| TSC22D3 | -1.538 | 5.22E-05 |
| MID2 | -1.5823 | 0.004 |
| IL13RA2 | -6.843 | 5.59E-05 |
| GRIA3 | -1.3238 | 0.0079 |
| BGN | -6.0805 | 3E-05 |
| MPP1 | -1.5478 | 0.0072 |
